# Cryo‐EM structures of multiple‐peptide resistance factor (MprF) from *Pseudomonas aeruginosa*

**DOI:** 10.1111/febs.70519

**Published:** 2026-05-22

**Authors:** Shaileshanand Jha, Kutti R. Vinothkumar

**Affiliations:** ^1^ National Centre for Biological Sciences, Tata Institute of Fundamental Research Bengaluru India

**Keywords:** aminoacylation, cryo‐EM, flippase, multiple‐peptide resistance factor

## Abstract

Aminoacylation of the lipid head group in many bacteria is carried out by bi‐functional enzymes called MprF, which encode a soluble synthase domain that typically transfers lysine or alanine from a tRNA to lipid head groups. The modified lipid is subsequently translocated across the leaflets by a transmembrane domain. This modification of lipids probably evolved to adapt to the environment where the microbes reside. Here, we describe the cryo‐EM structures of MprF enzyme from *Pseudomonas aeruginosa*, revealing a dimeric enzyme with a distinct architecture when compared with the homologous *Rhizobium* enzymes, and validate this arrangement with biochemical analyses. The cryo‐EM maps and the models in detergent micelle and nanodisc reveal a conformational change of the terminal helix of the synthase domain, highlighting the dynamic elements in the enzyme that might facilitate catalysis. Several lipid‐like densities are observed in the cryo‐EM maps, which might indicate the path taken by the lipids, coupling the function of the two domains. The structures allow postulation of the binding modes of tRNA and lipid transport, and suggest that the mobile secondary structural elements in the synthase domain might play a mechanistic role in these functions.

## Graphical abstract

Multiple peptide resistance factor (MprF) is a bacterial enzyme that carries out the aminoacylation of lipid head groups. CryoEM analysis of MprF from *Pseudomonas aeruginosa* reveals a dimeric enzyme, distinct from its homologues in *Rhizobium*. The soluble GNAT domain contains a dynamic amphipathic helix (TTH) that adopts different positions in detergent micelle and nanodisc, relative to the membrane. The dynamic elements in the soluble domain might play an important role in accommodating substrates and in the transfer of amino acid to the lipid head group.

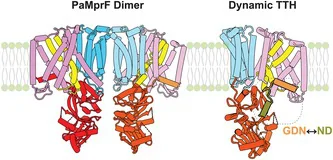

**Abbreviations**
Ala‐PG: alanyl‐phosphatidylglycerol
AMP: antimicrobial peptide
BMOE: bismaleimidoethane
BN‐PAGE: blue native polyacrylamide gel electrophoresis
CAMP: cationic antimicrobial peptide
cryo‐EM: cryogenic electron microscopy
DDM: *n*‐dodecyl‐β‐D‐maltoside
DM: *n*‐decyl‐β‐D‐maltoside
EM: electron microscopy
FSC: Fourier shell correlation
GDN: glyco‐diosgenin
GNAT: GCN5‐related *N*‐acetyltransferase
IMAC: immobilised metal affinity chromatography
Lys‐PG: lysyl‐phosphatidylglycerol
MprF: multiple peptide resistance factor
MRSA: methicillin‐resistant *Staphylococcus aureus*
MSP: membrane scaffold protein
PaMprF: *Pseudomonas aeruginosa* MprF
PG: phosphatidylglycerol
SDS‐PAGE: sodium dodecyl sulfate‐polyacrylamide gel electrophoresis
TM: transmembrane helix
TMD: transmembrane domain
tRNA: transfer RNA
TTH: terminal transferase helix
WT: Wild Type

## Introduction

The role of transfer RNA (tRNA) as an adaptor molecule in translation is well studied and beyond this more common role, charged tRNAs also fulfil other roles such as donating amino acids to lipids or carbohydrates and, in bacteria these form important processes in cell wall biosynthesis and antibiotic resistance [1, 2]. One such role for tRNA is found in the aminoacylation of lipid head groups, which not only allow microbes to adapt to the environment but also to counter the activity of small cationic peptides produced by a number of organisms [3, 4, 5]. To survive and grow in a competitive environment, organisms, in particular microbes, produce a number of small molecule metabolites that act on various cellular processes. One of these molecules constitutes the small peptides, which use the common property of the charge on the cellular membrane to kill other cells. These are collectively called cationic antimicrobial peptides (CAMPs), which are released by the host, including eukaryotes. They bind to the membranes and also promote pore formation [6, 7, 8].

Naturally, microbes develop resistance to such molecules and one of the resistance mechanisms to CAMPs in several pathogenic microbes, as well as some archaea, is mediated by a bifunctional enzyme called multiple peptide resistance factor (MprF). The family of MprF enzymes typically have two domains. The first, a cytosolic synthase domain allows binding of a charged tRNA and transfer of amino acid—typically a lysine or alanine (or arginine)—to the head group of a lipid on the inner leaflet [9, 10]. The second, a transmembrane (TM) domain, performs the flipping of the lipid to the outer leaflet to introduce modified lipid on the membrane surface, thereby rendering the CAMPs to be ineffective [3, 10, 11].

The lipid molecule that is commonly transferred with an amino acid is phosphatidyl glycerol (PG) but cardiolipin (CL) can also act as a substrate. MprFs are diverse enzymes both in their substrate specificity and the product generated. The enzyme from *Corynebacterium glutamicum*—which synthesises alanyl diacylglycerol using AlaDAGS [12], and LysX from *C. pseudotuberculosis*—which is responsible for the synthesis of lysine PG, and lysine diacyl glycerol, are all extremely specific [13], while LysPGS from *Listeria monocytogenes* transfers lysine to PG as well as to CL [14]. MprF homologues from a few other bacteria are promiscuous in transferring different amino acids to lipid molecules. These include MprF from *Bacillus subtilis*, which uses ala‐tRNA as well as lys‐tRNA as substrates for aminoacylation, and MprF2 from *Enterococcus faecium* that transfers arginine to PG in addition to alanine and lysine [15]. In contrast, *Clostridium perfringens* has two separate enzymes for synthesising AlaPG and LysPG [16]. When these MprFs are expressed in heterologous expression systems such as *E. coli* (where this enzyme is absent), modified lipids are synthesised and have been shown to decrease the effect of CAMPs, to provide resistance against antibiotics such as daptomycin, β‐lactams, as well as aminoglycosides [17, 18]. In addition, there are reports of several gain of function mutations that have been acquired in antibiotic resistant strains of various pathogenic microbes, including methicillin‐resistant *Staphylococcus aureus* (MRSA) [4, 19]. Thus, characterisation of these enzymes is interesting in many aspects, including the nature of recognition of the specific tRNA by the synthase domain, the mechanism of transfer of amino acid to the lipid head group as well as lipid transfer from one leaflet to the other.

Structurally, the soluble synthase domains from *Pseudomonas aeruginosa* and *Bacillus licheniformis* were the first to be characterised about a decade ago, revealing two GCN5‐related N‐acetyltransferase (GNAT) folds in tandem. Additionally, the bacillus enzyme was solved with substrate analogue L‐lysine‐amide, identifying a potential substrate‐binding site [20]. The structure of a close homologue FemX from *Weissella viridescens*, another ala‐tRNA transferase involved in peptidoglycan synthesis, revealed the peptidyl RNA conjugate‐binding site [21]. Together, these studies have been used to propose the possible binding sites for the aminoacylated acceptor stem arm of tRNA and a possible cavity for binding of the PG molecule. In 2021, the full‐length MprF structure of *Rhizobium tropici* by cryogenic electron microscopy (cryo‐EM) as well as the synthase domain by X‐ray crystallography was determined [22] and another cryo‐EM structure from *Rhizobium etli* has been deposited (EMD‐31445). These structures provide information on the relative position of the membrane and the soluble domain, the buried lipid molecules in the flippase domain and the possible mechanism of amino acid transfer and lipid flipping from the inner to the outer leaflet. Perhaps the interesting features of the full‐length structures from *Rhizobium sp*. are the arch‐like assembly of the monomers, the dimeric interface and the flexibility of the soluble domain. These enzymes’ diversity in substrates and products and importance in resistance to peptide‐like drugs prompts further characterisation of homologues from other organisms to understand their architecture and substrate specificity.

Here, we describe cryo‐EM structures of MprF from *Pseudomonas aeruginosa*, a well characterised enzyme that synthesises AlaPG [23], which lacks a positive charge but confers resistance to CAMPs as well as various other anti‐microbial compounds [16, 17, 24]. The structures of PaMprF determined both in detergent micelle as well in nanodisc reveals a more compact dimer and a distinct dimer interface with respect to the homologous *Rhizobium* structures. Biochemically, we show that the dimer interface observed in PaMprF is preserved in the membrane and the choice of the detergents affects the stability of the enzyme. Few lipid‐like densities are observed in the core of the flippase domain, and along with a number of other partially or fully ordered lipid molecules in the membrane domain, provide an explanation for the path taken by the lipids before or after modification to be assimilated with bulk lipids.

## Results

### Cryo‐EM maps of PaMprF in detergent micelle and nanodisc

**Fig. 1:**
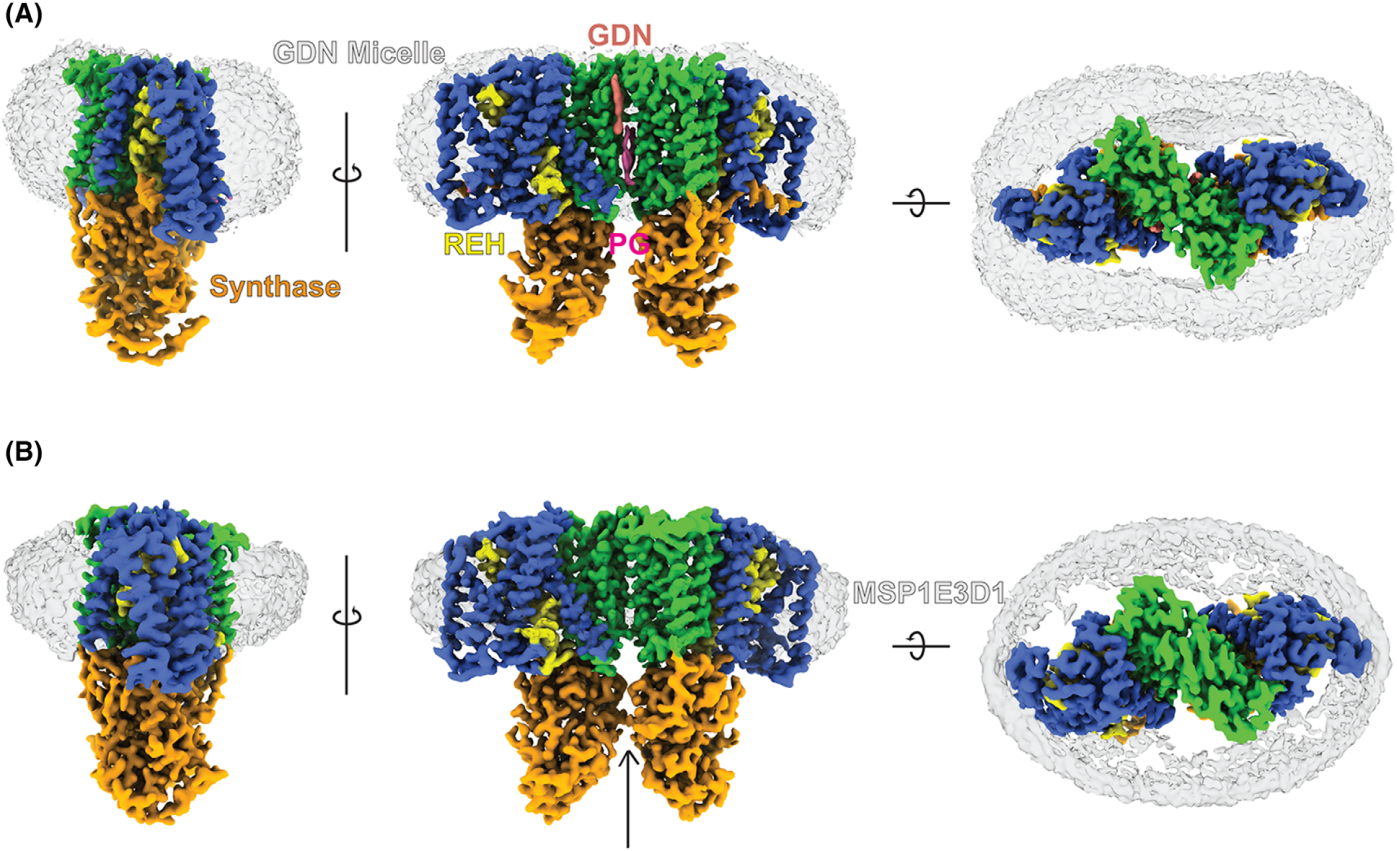
Cryo‐EM maps of PaMprF. (A, B) Different views of the cryo‐EM maps of PaMprF in glyco‐disogenin (GDN) micelle and nanodisc respectively. The central panel shows the front view and the dimer interface. On the left, the side view of the enzyme and on the right, the view from the periplasm is shown. The arrow in panel B shows the proximity of the soluble domain and the compact nature of the enzyme in nanodisc. The cryo‐EM maps are coloured, based on the functional regions of the enzyme, and the model was used to define the boundary. The peripheral helices, dimer interface and re‐entrant loops in the transmembrane domain (TMD) are coloured in blue, green and yellow, respectively. The soluble domain is coloured in orange. The detergent micelle and the nanodisc/lipid are shown as grey transparent surfaces around the TMD. The interface lipid and detergent at the dimer interface are shown in pink and salmon. In the periplasmic view, the continuous diffuse density in the GDN map and contrasting nanodisc belt can be clearly seen. The maps for the figures were generated using EMReady [29]. To show the detergent/lipid belt in both panels, a second map with the macromolecule removed is overlaid and the figures were made with chimerax [30].

The polypeptide of PaMprF was expressed and purified as a fusion protein with a cysteine protease domain (CPD) and a His‐tag [25]. The tag was cleaved by inducing the CPD activity with inositol‐6‐phosphate (IP_6_). Extraction of the enzyme from the membrane with lauryl maltose neopentyl glycol (LMNG) and subsequent exchange to glyco‐diosgenin (GDN) provided the best profile in size‐exclusion chromatography. Peak fractions were pooled and concentrated, and this sample was used for cryo‐EM studies. Reference‐free 2D classification of PaMprF in GDN revealed a dimeric enzyme with different views and a general flexibility of the soluble synthase domain (Fig S1A,B). Multiple rounds of 3D classification in RELION and heterogenous refinement in cryoSPARC [26, 27, 28] were used to enrich the population of particles that had well‐resolved transmembrane (TM) helices (Fig. S1C,D). This revealed two major populations, one where both the soluble synthase domains were observed and the other where only one of the soluble domains was ordered (Fig. S1C). The particles from these populations were refined and reconstructed without the application of symmetry or with C2 symmetry for the particles with both the soluble domains ordered (Fig. 1A and Fig S1C,E, Table 1). As expected, the local resolution of the map reveals higher resolution at the core of the dimer, the peripheral TM helices at ~ 4 Å, and the soluble domain between 3.5 and 5 Å with well‐resolved regions at the membrane interface (Figs S1F and S4A). The reconstructions of the PaMprF with ordered soluble domains in both monomers using C1 or C2 resulted in similar resolutions (3.4 Å and 3.3 Å, respectively) with no major difference in the structure, and we have used the C2 symmetrised map for the large part of the analysis (Fig. S1 and Table 1).

**Table 1:** Data collection and model refinement statistics.

Data collection and model refinement						
	PaMprF GDN	PaMprF GDN	PaMprF ND	PaMprF H386C/F389C, ND	PaMprF, H566C, ND	PaMprF, H566C, GDN
PDB ID	9J8R	9J8Q	9J8S	9W50	9W51	
EMDB ID	61239	61238	61240	65648	65649	
Microscope	Titan Krios (300 kV)					
Detector	Falcon 3 (counting mode)					
Pixel size (Å)	1.07	1.07	1.07	1.07	1.07	1.07
Dose rate (e^−^/pixel/s)	0.55	0.55	0.49	0.51	0.57	0.58
Total dose (e^−^/Å^2^)	~29	~29	~26	~27	~30	~30
Defocus range (μm)	−1.5 to −3	−1.5 to −3	−1.5 to −3	−1.5 to −3	−1.5 to −3	−1.5 to −3
Initial number of particles	1,239,668	1,239,668	1,701,373	892,429	1,434,841	808,820
Final number of particles	109,157	109,157	88,069	104,195	131,805	96,372
Map resolution FSC 0.143 (Å)	3.4	3.3	3.5	3.3	3.2	3.5
Map sharpening B‐factor (Å^2^)^a^	−131	−124	−112	−123	−116	−135
Symmetry imposed	C1	C2	C2	C2	C2	C1
**Model Composition**						
Number of Chains	2	2	2	2	2	
Protein (# atoms)	6145	6145	6158	6149	6171	
Ligand (# atoms)	383	383	79	102	14	
Model resolution (FSC 0.5) (Å)	3.6	3.5	3.7	3.5	3.6	
**Average B‐factor** (**Å** ^**2**^)						
Overall	170.7	146.5	155.5	142.6	143.4	
Protein	170.9	146	155.2	142.9	143.4	
Ligand	170.1	153.6	177.9	129.8	134.3	
**RMSD**						
Bonds (Å)	0.007	0.007	0.006	0.007	0.007	
Angles (°)	0.9	1.0	1.0	1.04	1.06	
**Validation**						
Ramachandran plot (favoured/allowed/outliers)%	96.2/3.7/0.1	96.2/3.7/0.1	95.4/4.5/0.1	96.1/3.8/0.1	94/5.9/0.1	
Rotamer Outliers (%)	1.3	1.6	0.2	1.1	0.8	
Clash Score	1.9	2.1	3	3	3	
Molprobity score	1.3	1.4	1.5	1.4	1.5	

^a^ Different B‐factor sharpened maps and deepEMhancer/EMready maps were used during model building. The values indicated are from the postprocess step in RELION.

**Fig. 2:**
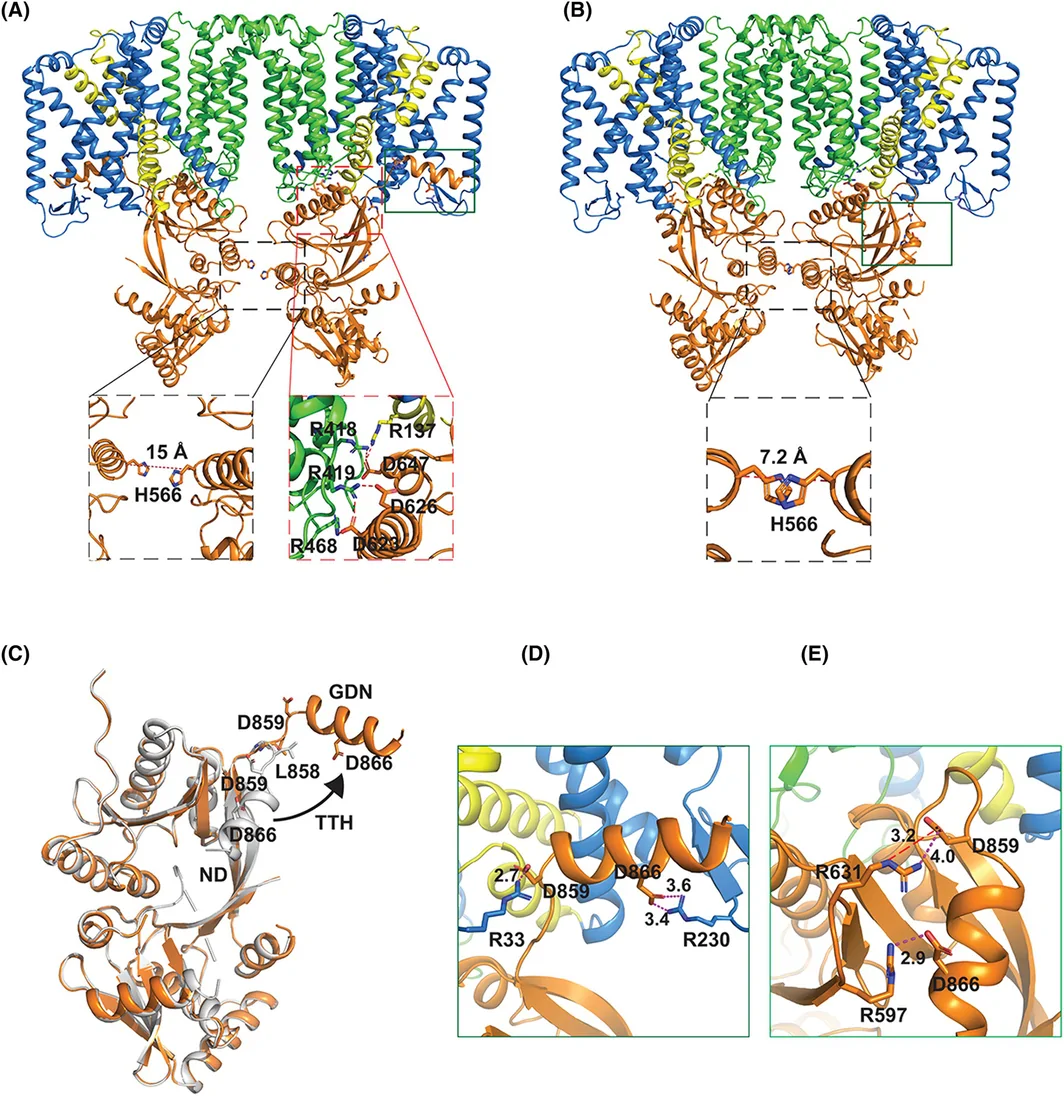
Models of PaMprF. (A, B) – models of PaMprF in glyco‐disogenin (GDN) micelle and nanodisc, respectively. The different regions of the molecule are coloured as in Fig. 1. The inset (black dashed line) shows the distance between the soluble domain in GDN and nanodisc as measured between the Cα of the H566 residue. In addition, the electrostatic interaction at the interface of the soluble and the TM domain (marked in red dashed line) between D623 and R468, D647, R137 and R418, and D626 and R419 are highlighted. (C) Overlay of the soluble domains of PaMprF in GDN (orange) and nanodisc (grey), respectively. The position of the terminal helix towards the membrane is indicated by the black arrow. (D, E) The interaction of terminal transferase helix (TTH) in GDN and nanodisc models, respectively. The residues D859 and D866 from TTH interact with the R33 and R230, respectively, in the model derived from the GDN EM maps. The same residues in the TTH of the nanodisc model interact with R597 and R631 in the soluble domain. The position of the TTH is marked with a green rectangle in the models shown in panel A and B. Figures were made with pymol [32].

To rule out the possibility of the CPD and its cleavage (by induction with IP_6_) causing any inadvertent effect, a 3D map of PaMprF was also obtained from a different construct where the cysteine protease domain was replaced with a precision protease site. This showed an identical architecture, indicating that the flexibility of the synthase domain is an inherent property of the enzyme in detergent micelle (Fig. S2). This further prompted us to reconstitute the enzyme into the MSP1E3D1 nanodisc with *E. coli* polar lipids (Fig. S3A), and a cryo‐EM map with C2 symmetry at an overall resolution of 3.5 Å was obtained with slight anisotropy (Fig. 1B and Figs S3B–G and S4B, Table 1). The belt‐like feature of major scaffolding protein (MSP) is clearly visible in the map (transparent grey surface in Fig. 1B). The enzyme in the nanodisc is more compact, and the soluble domain is better resolved than the enzyme in the GDN micelle (Fig. 2A,B). This is also supported by asymmetric reconstruction, which shows no large difference (Fig. S3D). When the maps of the dimer from GDN and nanodisc are compared, it is apparent that the axis of one monomer appears to be tilted with respect to the other monomer. Nanodiscs are primarily assumed to mimic the native lipid environment, but they are also known to constrict the TM helices depending on the size of the disc formed [31]. We observe a similar compaction with PaMprF in the MSP1E3D1 nanodisc, and these changes in the TM region can also be translated to the soluble domains.

### Structure of PaMprF

The model of the soluble synthase domain (PDB – 4v35) of *P. aeruginosa* was docked into the cryo‐EM maps, and the TM helices were initially built as a poly‐alanine model followed by assignment of the sequence. The fit of the models with the density of both the GDN and the nanodisc is shown in Fig. S4A,B. There are a total of 14 TM helices with two of them (TM3 and TM7) forming a pair of re‐entrant helices facing each other at the centre of the membrane (Fig. 2A,B, Figs S4 and S5A). The monomers from both GDN and nanodisc align well with an RMSD of ~ 0.3 Å for the TMD and some major differences in the soluble domain (discussed below). Comparison of the complete dimer model of GDN micelle and nanodisc reveals an overall RMSD of ~ 3.3 Å (Cα atoms) and a shift of 6.7 Å with respect to the centroid of the reference monomer (Fig. S5B). This can be appreciated with the proximity of the soluble domain in both the structures as measured between the residue H566 in the monomers (Fig. 2A,B). As expected, the volume of the cavity comprising the dimer interface is larger, 13089.1 Å^3^ in the GDN micelle as opposed to 8434 Å^3^ in the nanodisc.

The dimeric interface of the PaMprF is formed by TM 11 and 12, ECH4, and lipid molecules (modelled as phosphatidylglycerol [PG]), and a detergent molecule fill the interface (discussed below). The synthase domain is connected to the TM domain through a loop between the last TM 14 and the first helix of the first GNAT1 fold (Fig. S5A). Of the two GNAT folds in the synthase domain, the one closer to the membrane is better ordered (Figs S1F and S3F). There are potential electrostatic interactions between the synthase and the TM domain (Fig. 2A), two of these are formed at helix 3 of the GNAT1 fold by D623 and D626 with R419 in TM10 and D623 with R468 in the loop between TM 12/13. The third one is formed by the re‐entrant helix 3b (R137) and R418 with D647 on the fourth helix of the GNAT1 fold (Fig. 2A). Several regions of the GNAT2 fold are poorly resolved or disordered in both maps (GDN and nanodisc), and these include the residues between G806 and N832 comprising the fifth α‐helix and the loop between the second and third α‐helices. In the cryo‐EM maps, there are unmodelled densities in the soluble domain, membrane domain as well as at the interface between the membrane and soluble domain (Fig. S4C–F). These include an additional tubular density in PaMprF in GDN, which could be the fifth α‐helix of the second GNAT2 fold, and a density in PaMprF in nanodisc at a position similar to the terminal transferase helix (TTH) in GDN along with non‐protein densities, potentially lipid molecules, found more prominent in the GDN maps (Fig. S4C–F).

The last helix of the GNAT2 fold of the soluble domain, which we call the terminal transferase helix‐TTH (the soluble domain is called synthase, but its major role is in the transfer of aminoacid from tRNA to a lipid head group so it can also be termed transferase), occupies different positions in the maps obtained with GDN and nanodisc. In the model derived from the GDN map, the TTH is held in a parallel orientation to the membrane via the hydrophobic interactions of the non‐polar residues, as well as electrostatic interaction (Fig. 2C,D). Residues D859 and D866 in the TTH interact with R33 in TM1 and R230 in the β‐sheet connecting peripheral helices TM5 and TM6, respectively (Fig. 2D). In contrast, in the model derived from the nanodisc map, the TTH adopts a similar position to that observed in the crystal structure of the PaMprF soluble domain alone, facing towards the solvent and interacting with the first β‐sheet of the second GNAT fold (Fig. S5C). In this conformation also, there are interactions of the acidic residues (D859 and D866) with the R597 and R631 (Fig. 2E). An overlay of these models shows that the TTH moves as a rigid body with G857 acting as a pivot. The distance measured from the Cα distance of L858 between the two structures is ~ 5 Å (Fig. 2C). The different positions of the TTH in the two maps is a salient feature and might play a role in the mechanism.

### Comparison of MprF structures

**Fig. 3:**
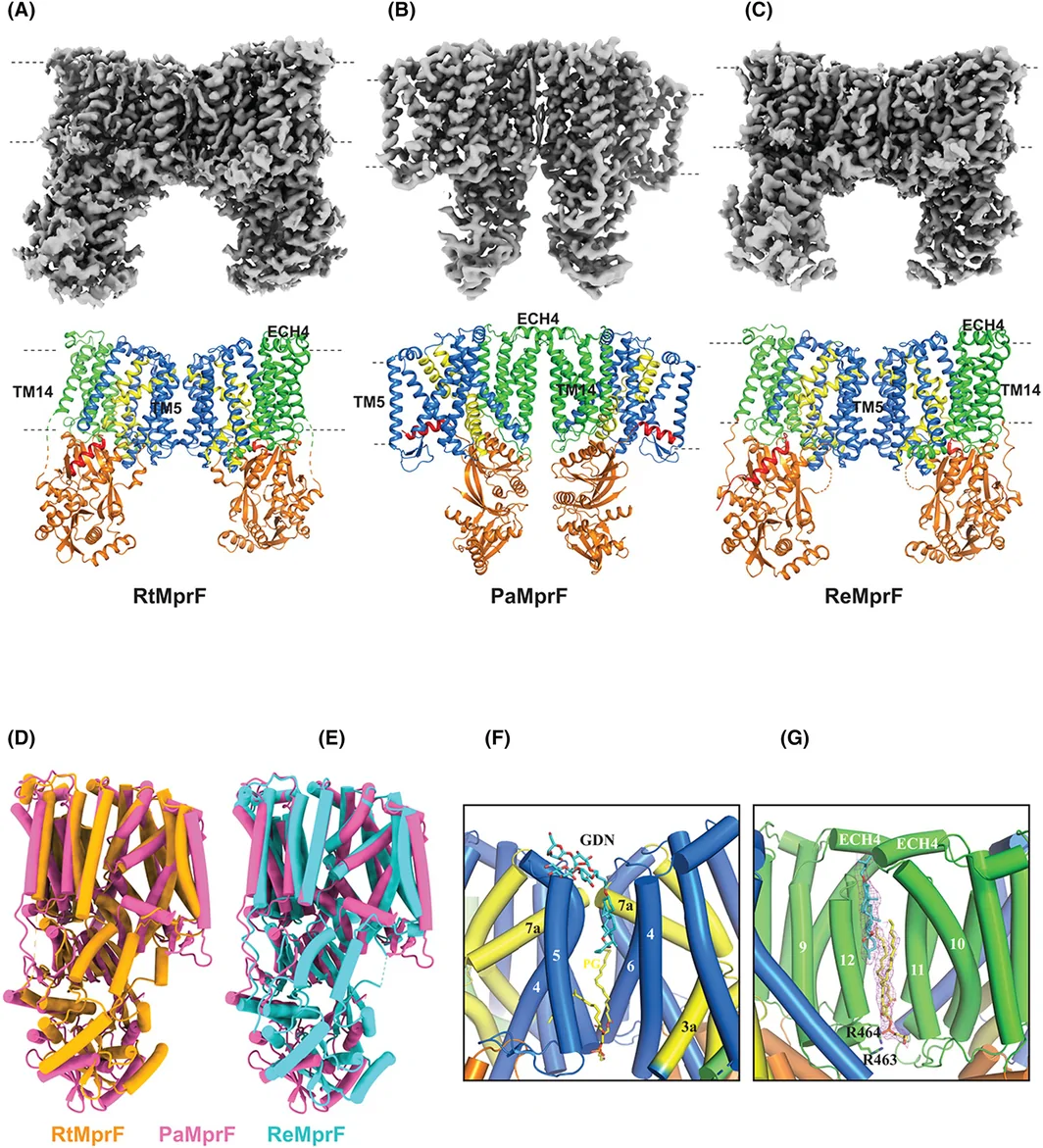
Comparison of MprF structures. (A–C) Comparison of the structures of different MprF homologues. The maps (top) and the models (bottom) of MprF from *Rhizobium tropici*, EMD‐30869 and PDB–7DUW (left), *Pseudomonas aeruginosa* in GDN (centre) and *Rhizobium etli*, EMD‐31445 and PDB–7F47 (right) are shown. The probable membrane boundary is marked by dashed lines. The models are coloured as in Fig. 1 with the N‐terminus of the transmembrane (TM) region in blue, re‐entrant helices in yellow and C terminal TM helices in green. The soluble domain is coloured in orange and the TTH in red. The difference in the dimer interface is clearly visualised and ~ 180° rotation of the monomers (position of the ECH4) relates to the dimer architecture. (D) and (E) Overlay of PaMprF monomer in GDN (magenta) with monomers of RtMprF (orange), and ReMprF (cyan) showing larger deviation in the membrane domain. (F) and (G) show the dimer interface of RtMprF and PaMprF, respectively. The detergent GDN is found in a similar position in both RtMprF and PaMprF despite the dimer interface formed by different TM helices. The dimer interface in RtMprF is formed by TMH 4, 5, 6, and 7a. In PaMprF, TMH 10, 11, 12 and ECH4 form the dimer interface. In panel G, the density around GDN and the lipid molecule is shown in magenta. Figures were made with chimerax and pymol [30, 32].

Two structures of MprF from *Rhizobium tropici* and the map, as well as the model for *R. etli* are available (PDB‐7F47 and EMD‐31445) [22]. The EM maps from *R. tropici* were obtained in nanodisc with the enzyme purified either in DDM or GDN (but the initial solubilisation in each preparation was performed with DDM). From the first glance, it was evident that the maps of *P. aeruginosa* and the *Rhizobium sp*. are different (Fig. 3A–C), which is obvious from the position of the soluble domain with respect to the TM domain as a result of different dimer interfaces (~ 180° rotation of the monomers). The monomeric models of these enzymes align with an overall RMSD for Cα atoms of ~ 4 Å for RtMprF and ReMprF (Fig. 3D,E). Not surprisingly, the interface and the salt bridges between the soluble and TM domains are also conserved in RtMprF. Greater deviation is observed in the TM domain of these structures (2.1 Å and 1.8 Å for RtMprF and ReMprF, respectively) when compared to PaMprF in GDN micelle, while the RMSD for the soluble domain alone is ~ 1 Å. The location of the helix corresponding to TTH in the structures of *Rhizobium* species is similar to the PaMprF (nanodisc) model, i.e., away from the membrane interacting with the soluble domain. In PaMprF, TM helices 11 and 12 and external helix (ECH4) (coloured green in Fig. 3B) form the dimer interface, while in both the *Rhizobium* MprFs, this is made up of TM helices 4, 5, 6 and 7a (coloured blue in Fig. 3A, 3C). The dimer interface in PaMprF is mainly hydrophobic in nature and the interface area is ~ 522 Å^2^, while in RtMprF, it is ~ 618 Å^2^ and includes two salt bridges (residues R222 and D220 of the opposite chain) along with hydrophobic interactions and hydrogen bonds. Perhaps an interesting feature is the position of the ordered lipid and detergent, which is found in similar positions in both the PaMprF and RtMprF maps despite the difference in the dimer interface (Fig. 3F,G). Thus, the difference in the oligomeric arrangement of MprF from different homologues is intriguing, might indicate an evolutionary feature between species and a functional relevance, or can alternatively be an effect arising from the choice of the detergent.

### Effect of detergents on PaMprF

In the initial stages of the PaMprF project, we performed a detergent screen with various detergents and lipids, both during solubilisation and purification. Solubilisation of the PaMprF from the membranes with N‐dodecyl β‐D‐maltoside (DDM) and exchange to other detergents during purification rendered the enzyme unstable (Fig. S6A). However, solubilisation with LMNG or digitonin and subsequent exchange to GDN provided the best profile in the Blue Native‐PAGE (BN‐PAGE) gel (as well as in the size‐exclusion chromatography). In hindsight, the importance of GDN can be appreciated by its localisation at the dimer interface (Fig. 3G). It was evident from BN‐PAGE that predominantly monomeric bands were observed when solubilisation was performed with DDM and subsequently exchanged to GDN (Fig. S6A,B). In contrast, when membranes were solubilised with LMNG and exchanged to GDN, a strong dimeric and some higher molecular weight bands are also observed (Fig. S6A,B). Thus, the first step of extraction from the membrane with the milder detergents such as LMNG or digitonin is essential for the oligomeric stability of PaMprF. As a consistent density of cholesterol skeleton from GDN was observed at the dimer interface in the EM maps of PaMprF from different biochemical preparations and also observed in the cryo‐EM map of RtMprF (Fig. 3F,G), we asked if cholesterol hemisuccinate (CHS) (commonly used in eukaryotic membrane protein purification but recently used for prokaryotic proteins [33]) can be added during solubilisation with LMNG or DDM to stabilise PaMprF (we note that cholesterol is not found in *E. coli* or *Pseudomonas*). It was observed that the presence of CHS during the solubilisation step improves the yield of PaMprF but does not remove the necessity of GDN during purification (Fig. S6C).

**Fig. 4:**
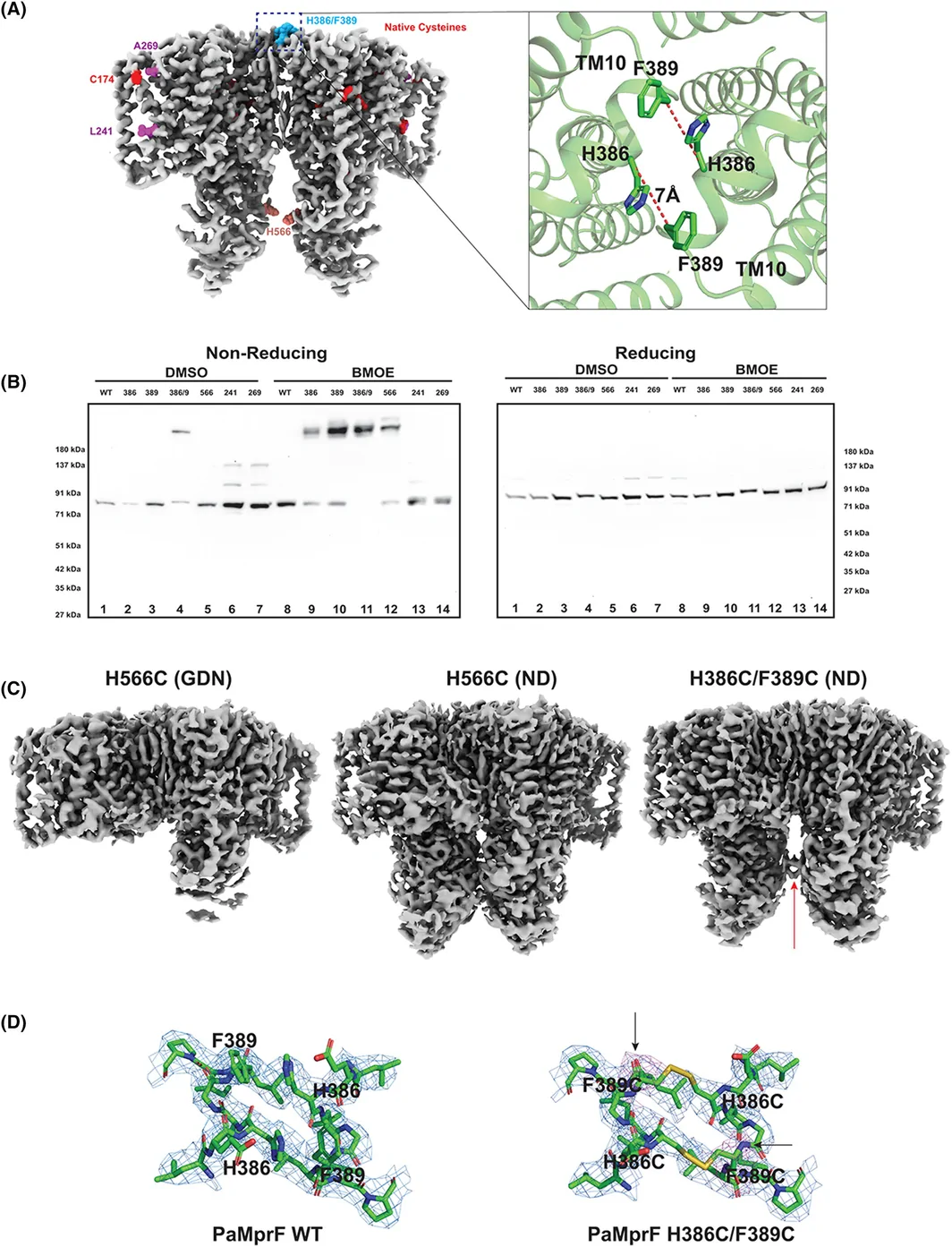
Analysis of dimer interface of PaMprF with cysteine cross‐linking and cryo‐EM. (A) Introduction of non‐native cysteines in PaMprF. Cryo‐EM map of PaMprF in glyco‐diosgenin (GDN) coloured in grey, and the position of the native cysteine residues coloured in red. The H386 and F389 residues at the dimer interface on the periplasmic side are coloured in cyan, L241 and A269 in magenta, and H566 in salmon. Note the proximity of A269 and the C174 in a possible *Rhizobium*‐like dimer interface. Inset of the dimer interface from the periplasmic side showing the α‐helices connecting the transmembrane domain (TMD) 9 and 10 in each monomer harbouring the residues H386 and F389. The Cα distance between the H386 of one monomer and F389 of the other is represented by a red dashed line and is ~ 7 Å. The figure was made with chimerax and pymol. (B) Cross‐linking of the cysteines on the *E. coli* membranes expressing PaMprF. The Western Blots showing cross‐linking of PaMprF with bismaleimidoethane. Lanes 1–7 correspond to the membrane samples of WT, H386C, F389C, H386C/F389C, H566C, L241C and A269C, respectively, with DMSO. Lanes 8–14 correspond to the same samples with cross‐linker BMOE added. The samples on the left and right blot are in non‐reducing (*n* = 3) and reducing conditions (*n* = 1, it was performed only once to make sure the higher molecular bands were formed via cysteines and are specific), respectively. Lane 4 in the non‐reducing blot shows that the H386/F389 migrates as a higher molecular weight band even without the addition of the cross‐linker, indicating the proximity of the residues in the membrane. The monomer of H386C/F389C migrates slightly slower when compared to wildtype and other mutants. 10 μg of total membrane protein was loaded in each lane. The inability of cross‐linking by BMOE under reducing conditions indicate that these oligomers formed are mediated by disulphide bonds. Note that no cross‐linking is observed in lanes 13–14 corresponding to cysteine mutants at the *Rhizobium*‐like interface of PaMprF. There are additional bands in these lanes, which are also observed in WT, and in reducing conditions, are unlikely to be disulphide‐linked as their intensity does not increase upon the addition of a cross‐linker. (C) Cryo‐EM maps of cysteine mutants of PaMprF. Reconstruction of PaMprF, H566C (GDN) left showing a commonly observed PaMprF structure in GDN with only one of the synthase domain‐ordered. Cryo‐EM map of the H566C in nanodisc (centre) showing a dimer interface and the location of TTH, similar to the WT nanodisc structure. Cryo‐EM map of H386C/F389C with BMOE added to the membrane prior to detergent solubilisation. The structure reveals a GDN‐like dimer interface in a nanodisc with the TTH location consistent with the WT nanodisc structure. An additional unmodelled density at the interface of two soluble domains is also observed. Figure was made with chimerax [30]. (D) Cryo‐EM density of dimer interface of PaMprF WT and H386C/F389C. Final sharpened maps were used to make this figure in pymol [32] and contoured at 7σ. In the H386C/F389C, it is likely that one of the cysteine (residue 389) is in an alternate conformation (density in magenta and marked with an arrow). In the current model, only the disulphide‐formed conformation is modelled (see also Fig. S11).

Although the nanodisc structure of PaMprF with lipids indicates the same dimer interface, the initial steps in the purification still involved the addition of detergent, and it can thus be argued that the observed arrangement of the monomers could be a result of some rearrangement during the process of solubilisation and reconstitution. To probe the organisation of the PaMprF monomers in the membrane, cysteine cross‐linking was performed. The enzyme PaMprF has a total of eight cysteine residues; seven of these are in the TM domain and one in the soluble domain (Fig. 4A). The loop connecting the TM 9 and 10 on the periplasmic side forms a short helix, which is antiparallel to its counterpart on the other monomer at the dimer interface. H386 of one monomer is ~ 7 Å away from F389 of the opposing monomer (the distance between the Cα atoms and shown in the inset in Fig. 4A). Both these residues were mutated to cysteine (H386C/F389C) to determine the proximity of these helices in the membrane environment (note that the native cysteines have not been mutated). Similarly, residue H566 in the synthase domain is closer in the dimer observed in PaMprF and can potentially form a cross‐link. We also introduced cysteines at two other residues, namely L241 and A269, in the TM domain, which, in a hypothetical *Rhizobium*‐like dimer model, would be positioned close to their counterparts on the opposing monomer. In this hypothetical model, A269C can also potentially form a double cysteine mimic in conjunction with C174 (Fig. 4A).

The membrane fractions of the WT and the cysteine mutants of PaMprF were analysed in non‐reducing conditions with anti‐strep antibody using two different cross‐linking reagents, bismaleimidoethane (BMOE, Cat# 22323; ThermoFisher‐Pierce, USA) with a spacer arm of 7 Å (Fig. 4B) and copper phenanthroline (CuPt, Cat# 362204; Sigma, USA and Fig. S7A). Even in the absence of a cross‐linker, a higher molecular weight band along with the monomer band of the H386C/F389C double mutant was observed and the monomeric band of the double mutant migrates slightly slower (Fig. 4B and Fig. S7A). With the addition of the cross‐linker, only the higher molecular weight band was observed, and this cross‐linking could be prevented in the presence of ß‐mercaptoethanol (Fig. 4B and Fig. S7A). Single cysteine mutants of residues—H386, F389 and H566—also showed a higher molecular weight band in the presence of cross‐linker, but in the WT, the L241C and A269C mutants, no higher molecular weight band was observed. One possibility is that these mutated cysteines of residues L241 and A269 are not accessible for the cross‐linker. The monomer of PaMprF migrates between the 71–91 kDa markers (calculated mass of polypeptide is 98.2 kDa), and a dimeric form is expected to be between 140 and 180 kDa but the cross‐linked oligomer migrates at ~ 250 kDa. The higher size in migration after cross‐linking could either be due to aberrant migration in SDS‐PAGE or a higher‐order oligomer than a dimer.

To validate the oligomeric nature of the higher molecular weight band, we purified the double mutant H386C/F389C and H566C in GDN and reconstituted it into a nanodisc. When the H385C/F389C was analysed by size‐exclusion chromatography, multiple peaks (along with the void peak) were obtained (Fig. S7B), and these were analysed by both SDS‐PAGE (Fig. S7C) and BN‐PAGE (Fig. S7D). In the SDS‐PAGE, the mutants in the presence of cross‐linkers showed a higher molecular weight band similar to that observed in the western blot (Fig. 4B and Fig. S7C). No shift in size for the WT and the mutant enzymes in BN‐PAGE was observed in the presence of a cross‐linker (Fig. S7D). When SDS was added to the sample before loading on the BN‐PAGE gel, a second band corresponding to a dimer both in GDN and in nanodisc was observed, but only in the mutants and in the presence of the cross‐linker (Fig. S7D). These observations thus indicate that the enzyme does form a dimer as observed in the cryo‐EM maps of PaMprF, and can dissociate easily in the presence of certain detergents.

We also performed 3D reconstruction of the cysteine mutants in the presence or absence of cross‐linkers to ascertain the oligomeric state and the dimer organisation (Figs S8, S9, and S10). The periplasmic double mutant—H386C/F389C—was cross‐linked with BMOE prior to detergent solubilisation, followed by purification and nanodisc reconstitution. The cryo‐EM structure revealed a dimeric assembly with a larger interface volume similar to the GDN‐purified WT PaMprF, but the TTH remained localised towards the synthase domain (Fig. 4C and Fig. S8). Interestingly, there is an ambiguous additional density between the two synthase domains that remains unmodelled (marked with an arrow in Fig. 4C). The interface density with the mutated cysteine and the disulphide bond is clearly observed in the map (Fig. 4D) but there is a possibility that some populations of the PaMprF have the cross‐linker bound (Fig. S11A,B). We also obtained reconstructions of the H566C mutant both in GDN and in the nanodisc. The image‐processing of the H566C in GDN reveals a flexible and partially ordered synthase domain similar to the WT enzyme in GDN (Fig. S9). This would also explain the lack of cross‐linked dimeric species in the non‐reducing SDS‐PAGE for H566C mutant in GDN (Fig. S7C). The cryo‐EM map of H566C in the nanodisc is similar to the WT PaMprF in the nanodisc and the synthase domains are better ordered, consistent with a significant dimeric population in the non‐reducing SDS‐PAGE even in the absence of any cross‐linker (Figs S7C and S10). However, in the current map, unlike the double mutant, the density for the disulphide is not prominent and hence not modelled (Fig. S11C).

### Lipid molecules in PaMprF

**Fig. 5:**
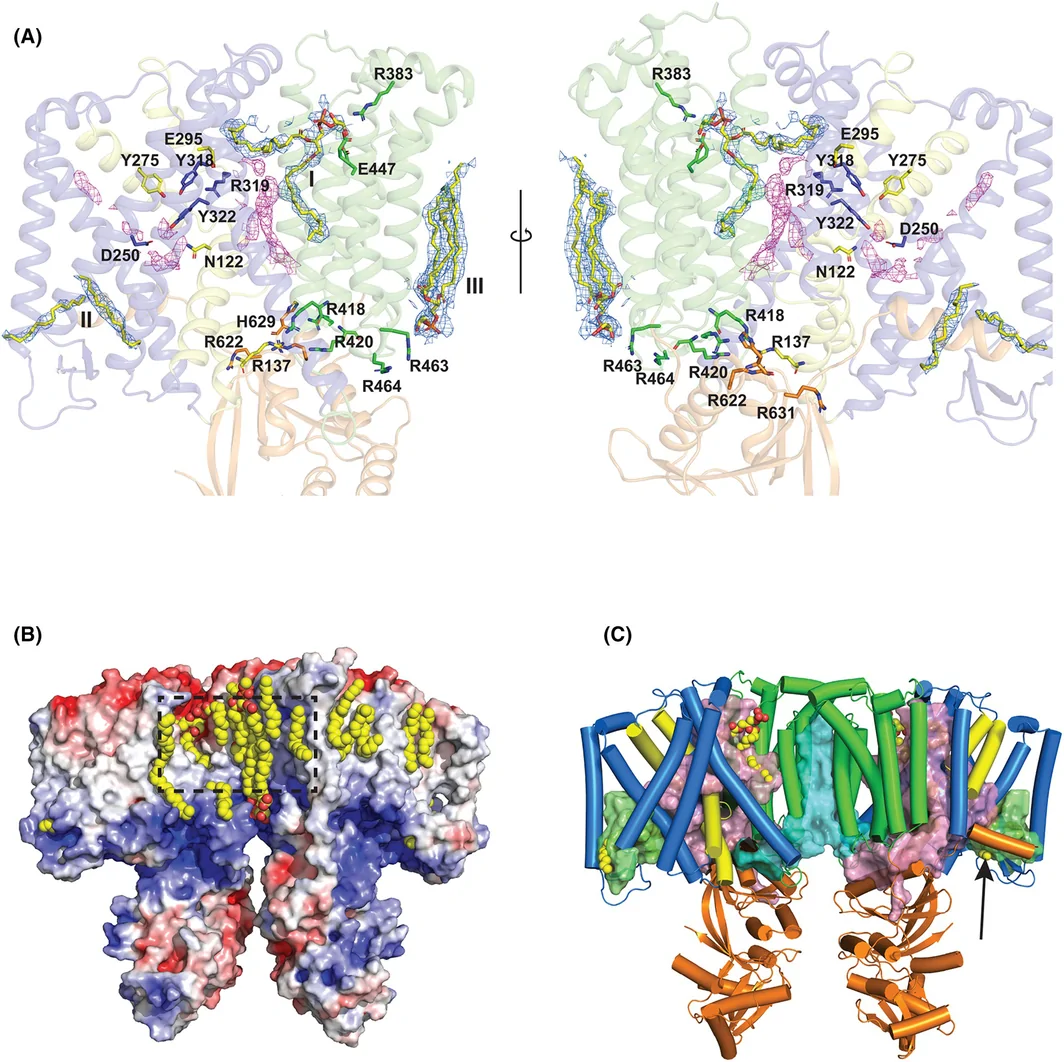
Lipid molecules in PaMprF in GDN micelle. (A) Two views of the membrane domain and the interface with the synthase domain are shown (coloured as in Fig. 1), with few salient lipid molecules in stick representation (carbon atom coloured in yellow). The sharpened cryo‐EM map (in blue mesh) at 5σ and carved 2 Å around the atoms is shown around the lipid molecules. Key residues that might play a role in lipid translocation are shown in stick representation. There are two partially ordered lipids close to TM4 and TM5 and one of them close to TTH (marked as II), whose head group is likely to be close to the soluble domain. There are fragmented densities on either side of the re‐entrant helices and are currently unmodelled (magenta mesh). One PG molecule (marked as I) is modelled close to the periplasmic side, and residues E447, E448 and R383 might mark the path for entry and exit of the lipids. Cluster of positively charged residues (R137, R418, R420, R463, R464, R622, and R631) line the interface between the membrane and the soluble domain and likely to play in interacting with lipid and or tRNA. The density for the lipids marked with I, II and III are also shown in Fig. S4F. (B) The electrostatic potential map of PaMprF is shown with lipids in sphere representation and coloured in yellow. The blue colour in the surface map represents a positive charge and red represents negative. The lipid molecules bind to the grooves and crevices of the enzyme. The lipid molecules that are lined up one after the other at the periplasmic side is marked with a dashed black rectangle. (C) The two cavities coloured pink and green that form the passage for the possible transport of lipids in the TM domain. The smaller cavity coloured green is formed by the peripheral helices and the TTH, while the larger one coloured pink is present in the centre of the TM domain, bifurcated by the re‐entrant helices (coloured yellow) into two subcavities. The cavity formed by the dimer interface is coloured cyan. Lipid molecules (sphere representation) are found in the cavity and the one close to TTH are marked with an arrow. Figures were made with pymol [32].

A number of non‐protein densities are observed in the cryo‐EM maps derived from the GDN micelle—some of them with the classical pose of lipids with two acyl lengths and others that reveal a short tube‐like density—have been modelled as single acyl chains of either PG or phosphatidyl ethanolamine (PE) (Fig. 5A,B, Fig. S4E,F). These densities attributed to lipid molecules were also verified by calculating a fofc omit map with servalcat [34]. The number of lipid‐like densities in the outer leaflet is greater in number, perhaps indicating a dynamic nature at the cytoplasmic leaflet (Fig. 5B). A series of single acyl chain densities is evident from one of the openings on the periplasmic side (marked in the rectangle in Fig. 5B), and in the surface representation it is evident that many of these lipid molecules fit into the grooves of the enzyme. Among the modelled lipid molecules, we would like to describe a few that might be of functional relevance (Fig. 5A,C). Two partially ordered lipid molecules are found close to TM4/TM5, and there are fragmented densities at either side of the re‐entrant helices TM3 and TM7 (Fig. 5A), which have not been modelled currently. But these unmodelled densities are close to key residues that might play a role in lipid translocation [19]. Of interest in the PaMprF structures are the presence of clusters of charged residues, including R137, R418, R419, R420, R463, R464, R468, R622, R631 and H629. These residues are positioned at the interface of the TMD and the synthase domain, and could play a role in interacting with acidic residues (Fig. 2A), lipid head groups, and perhaps, in their interaction with tRNA at the membrane/synthase domain interface. At the dimer interface, there is density for two lipid molecules at the inner leaflet, one completely ordered and the other partially ordered, and they are currently modelled as PG molecules but could also be cardiolipin (Figs 1A, 5B and Fig. S4F). R463 and R464 can form potential hydrogen bond interactions with lipids from the same as well as the opposite monomer. In addition, a lipid is modelled close to the periplasmic side, which is surrounded by TM1, TM8, TM9 and TM13, (Fig. 5C) and opening towards the periplasmic space. An equivalent density at the same position is also observed in the nanodisc and the H383C/F389C mutant maps, and it could be alanyl‐phosphatidylglycerol (Ala‐PG) but we do not have structural or biochemical evidence at the moment to support this and it is hence modelled as PG. This lipid is in close proximity to residues R383, E447 and E448 (Fig. 5A and Fig. S4F).

Analysis of the PaMprF enzyme for cavities reveals interesting features beyond the one observed at the dimer interface in both GDN and the nanodisc maps. The arrangement of the TM helices in PaMprF gives rise to a larger cavity encompassing the TM domains centred around the re‐entrant helix (coloured pink in Fig. 5C). Due to the movement of TTH in the GDN maps, this cavity has a slightly smaller volume (5365.1 Å^3^) than in the nanodisc (6017.7 Å^3^). This larger cavity can be divided into a sub‐cavity formed by TM helices 4, 5 and 6 and the β‐strands connecting TM5 and 6 and the TTH (coloured green in Fig. 5C), which is about 974 Å^3^ in volume and harbours a partially ordered lipid molecule perpendicular to the TTH, and co‐planar to the membrane surface (Fig. 5C). This cavity perhaps provides a path for holding lipids before or after the transfer of amino acid from tRNA. Taken together with partially or fully ordered lipids in the PaMprF, in particular those lined up towards the periplasmic side or the outer leaflet (Fig. 5B,C), and the vicinity to the cavities described above, this indicates the possible path taken by the lipids to enter and exit the enzyme.

## Discussion

Microbial resistance to small molecules such as antibiotics involved in translation inhibition can arise from small changes, for example, methylation of rRNA bases [35]. Similarly, the resistance to CAMPs that are widely produced by many organisms, can be  achieved by the transfer of an amino acid from tRNA to a lipid head group, a relatively simple solution using the charged tRNAs in the cells. Mechanistically, this is also interesting, as the protein that performs this function requires both a charged surface and a hydrophobic region for tRNA and a lipid molecule, respectively, to bind and bring the head group of lipids in close proximity to tRNA for the amino acid to be transferred. Additionally, the lipid with the modified head group needs to be translocated from one leaflet to other. Such dual‐functional enzymes are more commonly found in pathogenic bacteria conferring resistance to CAMPs, and several enzymes from multiple species have been characterised [3, 24].

In this study, we report the cryo‐EM structures of the PaMprF, both in GDN micelle and MSP nanodisc (Fig. 1). The maps reveal a dimeric enzyme as observed in the homologous *Rhizobium* enzymes [22]. While they share the same monomeric fold, they have different dimeric interfaces (Fig. 3). In PaMprF, the dimeric interface is mediated by ECH4, TM11 and TM12, and in *Rhizobium* enzymes this interface is largely formed by TM4, TM5, TM6 and TM7a (Fig. 3A–C). Recently, a monomeric structure of PaMprF bound to a sybody purified in DDM and reconstituted in a saposin nanodisc was also reported [36]. The origin of these differences is intriguing and might represent different states of the enzymes or the effect of detergents used in the process of extraction and purification.

When PaMprF was screened with detergents in different combinations and analysed by BN‐PAGE, it was evident that use of DDM for initial solubilisation made the enzyme less stable and LMNG was required (Fig. S6A). A subsequent exchange to GDN kept the enzyme in an oligomeric state only if extracted initially with LMNG or digitonin (Fig. S6A,B). Further, reconstitution of PaMprF into a nanodisc revealed a dimeric assembly similar to that in the micelle (Figs 1 and 2). Cross‐linking experiments with engineered cysteine residues at the oligomeric interface of PaMprF, either at the periplasmic or the cytosolic side, revealed a dimeric enzyme in the micelles, and also in the membrane (Fig. 4B and Fig. S7A,C,D).

Interestingly, in most of the datasets of PaMprF, either in detergent or nanodisc, we observed particles that are different from those classes with more populated particles and which classify as separate classes (Fig. S12). These primarily include top views of a tetrameric assembly of PaMprF (WT and H566C) or top and top‐tilted views of a larger circular nanodisc density. The most notable among these are the side views of the WT and H566C PaMprF datasets in nanodisc, which look similar to the classes corresponding to the *Rhizobium* dimer‐like interface (Fig. S12). The two soluble domains are placed far apart, and the dimer interface forms a depression in the centre of the periplasmic leaflet, unlike the usual upward‐pointed periplasmic interface region. These classes correspond to only <1–2% of the particles obtained after initial rounds of 2D classification, and hence a high‐resolution reconstruction could not be achieved for this subset of particles (Figs S10 and S12). These observations support that the oligomer of PaMprF is extremely labile and can adopt a different dimer interface upon detergent extraction and reconstitution in a nanodisc. GDN can help in maintaining such an oligomeric state as recently shown for MgtA, a magnesium transporter where a dimeric reconstruction could be obtained in GDN but a largely monomeric population was observed in DDM and LMNG. Notably, in MgtA the dimerisation was mediated largely by the soluble domain [37].

Sequence analysis of MprF enzymes reveals very little conservation in regions contributing to either PaMprF‐like or *Rhizobium*‐like interfaces (Fig. S13). Interfacial lipids have been implicated in stabilising the oligomeric states of membrane proteins, and depending on the detergent used, it is noted that the oligomeric state can change [38]. Thus, we conclude that the MprF architecture/oligomeric state is sensitive to the type of detergent used, both during initial solubilisation and in reconstitution into nanodisc. The arrangement of monomers as observed in PaMprF in this study is realistic as revealed by cross‐linking in the membranes, and the functional importance of this oligomeric state in‐vivo remains to be studied (Fig. 4).

The synthase domain of PaMprF is structurally well characterised, and the mutagenesis of conserved residues has indicated that they play a role in specificity and interaction, and the substrates themselves play a role in catalysis [20, 39]. In the PaMprF GDN map, synthase domains reveal greater flexibility but are more compact in the nanodisc map (Fig. 2A). Of particular interest is the last C‐terminal helix in the synthase domain, which has previously been predicted to be a putative TM helix [15]. The deletion of this C‐terminus helix in PaMprF is known to abolish Ala‐PG synthesis and proposed to be important in substrate recognition [39]. The cryo‐EM map of PaMprF in GDN reveals that this helix can be buried in the membrane, explaining how the synthase domain alone is sufficient to synthesise modified lipids (Figs 1A and 2C) [39]. The two structures of PaMprF from detergent and nanodisc perhaps reveal two different conformations of TTH, and the two acidic residues—D859 and D866—from TTH in both the structures form electrostatic interactions with arginine residues, with the TM as well as the soluble domain (Fig. 2D,E). This switch of TTH between the two positions is a possible mechanism to allow the tRNA molecule to bind, and for the lipids to come closer to tRNA.

**Fig. 6:**
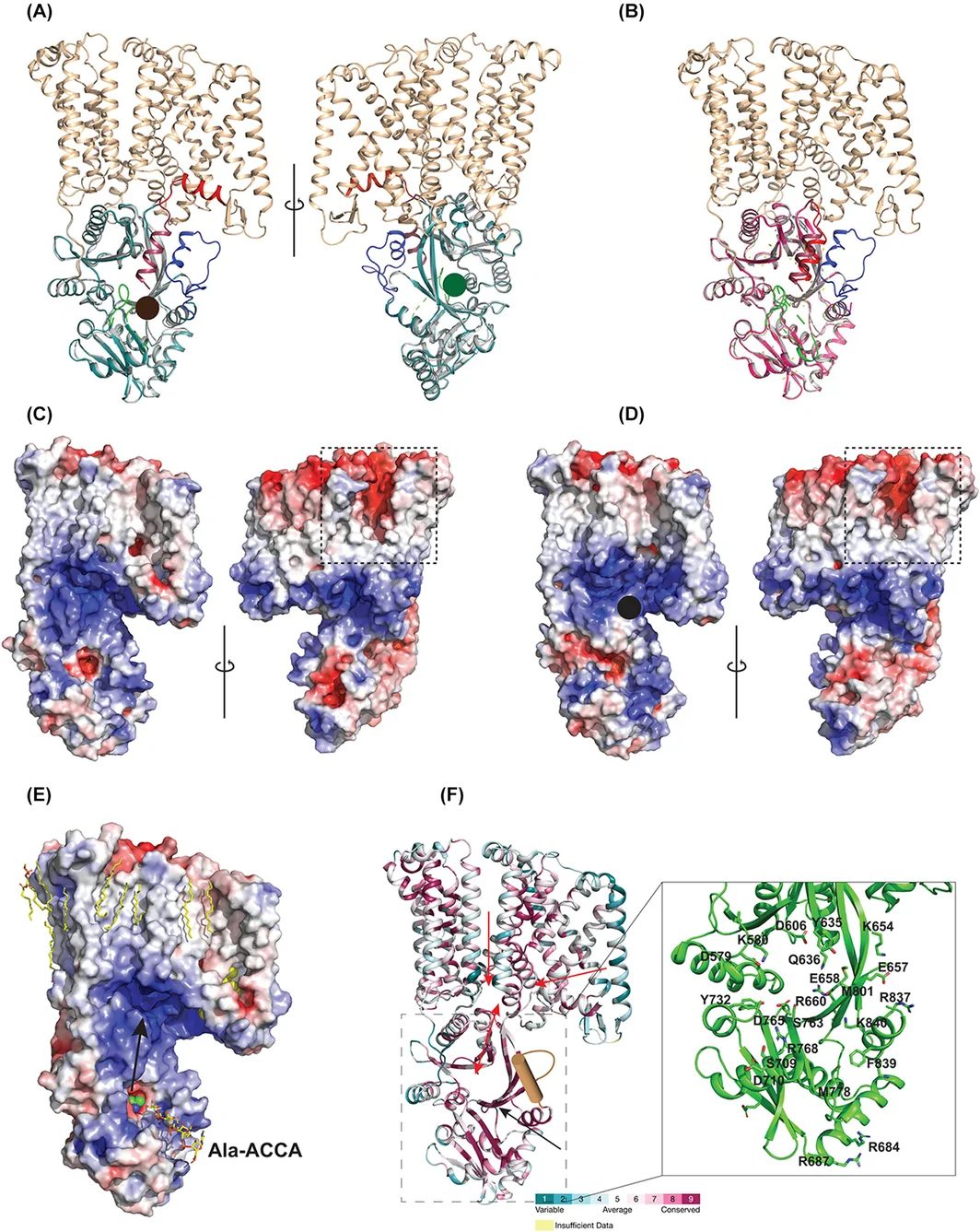
A model for tRNA‐binding and aminoacylation. (A) An overlay of the monomer model from the GDN cryo‐EM map and the soluble domain (PDB – 4v35). In the cryo‐EM GDN model, the TM domain is coloured in wheat, TTH in red and the synthase (soluble) domain in teal. In the crystal structure of the soluble domain, the TTH is coloured in brown, the helix 6 of GNAT2 in blue and disordered loop in green, while the rest of the molecule in grey. Two views of the molecule are shown. The cavities marked in brown and with a green circle are the postulated tRNA‐binding and the lipid binding sites, respectively [20]. (B) An overlay of the models of the nanodisc structure and the crystal structure of the isolated soluble domain (PDB – 4v35). The colouring of the crystal structure is the same as in panel A, the synthase domain of the nanodisc is in magenta and the TTH occupies a similar position. (C, D) Electrostatic surface representation of GDN and nanodisc monomer models. Two views are shown and coloured with −5 to +5 kT. The black dotted box shows the possible entry/exit site for the lipids at the periplasmic side and the outer leaflet (see also Fig. 4B). Note the general and continuous positive surface in the synthase domain and at the interface. In panel D, the black circle marks the position of TTH, which might shield the surface and prevent the binding of tRNA. (E) A simple docking exercise using ACCA‐ala in the predicted tRNA binding site (brown circle in panel A). The alanine residue fits snugly in the cavity within the synthase domain (also compare with panel C). However, there is no clear path for lipids to reach this site without exposing itself to the solvent. The black arrow might mark the path for the lipid aided by secondary structural elements in the synthase domain and can form a shield in front of it to interact with tRNA. (F) A monomer of PaMprF coloured according to the conservation of amino acid residues with Consurf [40]. The helix and the loop in the synthase domain that is unmodelled is shown in a cylindrical representation (wheat colour). The black arrow marks the possible interaction of the tRNA and the arrows in red mark the possible path for the lipid molecules to be placed and brought closer to the tRNA. A zoomed‐in view of the synthase domain with some conserved residues are shown (see also Fig. S13). Figures were made with pymol [32].

A previous structural study of the synthase domain (*Bacillus licheniformis*) with lysine amide that mimics the amino acid at the end of tRNA and a comparison with tRNA analogue‐bound FemX was used to extrapolate the tRNA binding site and conserved residues in the region, whose mutation resulted in reduction or loss of activity [20]. In that report, a lipid‐binding site opposite to the tRNA‐binding site was postulated as a tunnel that could accommodate a PG molecule and place it closer to the lysine amide (Fig. 6A,B). In the context of the full‐length PaMprF, the lipid binding site is exposed to solvent, and as previously observed in the RtMprF, there is no continuous tunnel or path from the membrane domain to the soluble domain that might protect the hydrophobic part of the lipid molecule. The electrostatic potential calculation of PaMprF shows a positive surface that extends from the cytosolic side of the membrane domain and the soluble domain (Fig. 6C,D). Extrapolating from the FemX‐bound partial tRNA analogue and UDP [21], a groove in the synthase domain is observed that can hold the tRNA (Fig. 6E). However, the membrane domain is further away, and there is no easy path for lipid to reach the tRNA binding site.

Among the MprF homologues, the regions of the soluble domain that face the tRNA binding are the most conserved (Fig. S13), and the presence of both the positive and hydrophobic residues can attract the aminoacylated tRNA as well the lipid molecules, and the binding of the substrates can push the TTH towards the membrane (Fig. 6F). The recent characterisation of the monomeric PaMprF with liposome transport assay defines this enzyme as a lipid scramblase transporting a wide range of substrates [36]. In addition, previous studies have shown that the specificity for the tRNA and the lipids are defined by the synthase domain and this is sufficient for generation of the modified lipids [22, 39]. Thus, with the available information in literature and from the structures presented here, we propose that the mobile elements of the soluble domain and the charge residues in the enzyme define the pathway for lipid molecules to be modified at the interface of the inner membrane and the cytoplasm.

In the models derived from the cryo‐EM maps of PaMprF, the loop connecting the second and third α‐helices, and helix 5 of the GNAT2 in the synthase domain are disordered and flexible, and the TTH was observed to adopt different conformations (Fig. 2A and Fig. S4C,D). Of these, the terminal C‐terminal helix, TTH might play two important roles. First, its movement towards the membrane allows for the tRNA‐binding and the head group of lipids to come closer to the synthase domain and possibly allow the helix 5 of GNAT2 to occupy this place (Fig. S4C), and secondly—by exposing hydrophobic residues—it forms part of the cavity where lipid molecules can be housed before and after amino acid transfer from tRNA (Fig. 5C and Fig. 6E). In the current maps, apart from the fragmented densities and partial density that can accommodate acyl chains near the inner leaflet, there is no strong evidence for a lipid molecule closer to the soluble domain (except at the dimer interface). The lipid molecule could be housed in the cavity (formed by TTH and TM4, 5 and 6, coloured green in Fig. 5C), and could approach the tRNA‐bound site with the changes created by the mobile elements. Upon modification with the amino acid, the modified lipid moves through the cavity formed by TMH1, 7, 8 and 11, with the re‐entrant helices facilitating the transport of the lipids using ionic interactions formed by its polar residues present at the loop region in these helices (Fig. S13). The cleft acts as the final exit site for the lipids that appear out of the sub‐cavities on the other side of a monomer and are then laterally moved towards the other monomer into these clefts arranged in a parallel array (Fig. 5C). Another hypothesis from the current structures and the presence of positive patches contributed from both the synthase and TM domains also suggest that it is possible for the tRNA to be closer to the inner membrane, which will be an elegant explanation but doesn't explain the specificity for the lipid head group for modification (Fig. 6C–E).

While the possibilities proposed here look like an attractive prospect, two caveats in the present study include the lack of a structure with tRNA or its analogue and an in vitro functional assay that can be used to measure both synthase and flippase activity simultaneously, and the physiological relevance of the oligomer. Nevertheless, the distinct oligomeric arrangement found between MprF from two homologues, structural changes in the GDN and nanodisc structures of the PaMprF, and the presence of lipids within the TM domain and on the periphery, provide an avenue to explore the mechanism of these bi‐functional enzymes.

## Materials and methods

### Protein expression and preparation of membranes

The MprF gene was amplified from the genomic DNA of *Pseudomonas aeruginosa* and cloned into the pET22b vector carrying a StrepTagII at the N terminus and a cysteine protease domain followed by a hexahistidine tag at the C terminus. A H386C/F389C double mutant was generated by a single pair of oligonucleotides using site‐directed mutagenesis [41]. All the constructs were verified by sequencing. For expression, the plasmids were transformed into *E. coli* BL21 (DE3) C41 cells [42]. A single transformant colony was inoculated in LB media containing 100 μg/mL ampicillin and grown overnight at 37 °C. This was used to inoculate larger cultures with 100 μg/mL ampicillin and allowed to grow at 37 °C till the OD_600_ reached 0.5. The temperature was reduced to 18 °C and induction was carried out by adding 1 mm IPTG for 16 h. All the following procedures were performed at 4 °C unless specified otherwise. The cells were harvested by centrifugation at 4000 g, resuspended in 100 mM Tris pH 8, 1 mm PMSF, 4 mM MgCl_2_, 0.5 mm CaCl_2_, 4 μg/mL DNase and 5 mm ß‐mercaptoethanol. Cells were lysed by passing the suspension thrice through Emulsiflex‐c3 (Avestin) at 15,000 psi. The unbroken cells were removed by centrifugation at 15,000 g for 30 min and supernatant was subjected to ultracentrifugation at 200,000 g in a Ti45 rotor (Beckmann Coulter) for 2 h to obtain the membrane fraction. The membranes were homogenised and resuspended with 50% wt/vol in a buffer A containing 50 mm Tris pH 8, 200 mm NaCl, 5 mm ß‐mercaptoethanol and stored at −20 °C. The total protein concentration in the membrane fraction was estimated using Bradford reagent [43].

### MprF purification in GDN

Membranes were thawed and diluted to a final protein concentration of 5 mg/mL using Buffer A containing 20 mM imidazole and solubilised using 1% LMNG at 4°C for 1 h and centrifuged at 150,000 g for 30 min to remove the insoluble fraction. The supernatant was then applied to a 5 mL HisTrap (GE Healthcare) column pre‐equilibrated with buffer A containing 0.006% GDN (buffer B) and 20 mm imidazole. The column was washed with 30 CV of buffer B containing 40 mm imidazole. Full length MprF was then eluted with buffer B supplemented with 200 mm imidazole. 200 μm IP_6_ was added to the eluate and incubated for an hour to cleave the CPD and the decahistidine tag before applying onto a 5 mL StrepTactin column (IBA) pre‐equilibrated with buffer B. The column was washed with 30 CV of buffer B and eluted using buffer B supplemented with 5 mm desthiobiotin. Purified MprF was concentrated using a 100 kDa cut‐off Amicon concentrator and injected onto a Superdex 200 Increase size exclusion column with buffer B as the mobile phase. The peak fraction was concentrated to 5 mg/mL and used for further experiments. The steps in the purification of PaMprF with HRV3C construct was essentially the same as above, with only a decahistidine tag instead of the CPD domain and incubating IMAC eluate with HRV3C protease instead of IP_6_.

### MSP1E3D1 purification

The membrane scaffold protein MSP1E3D1 was purified according to the previous published protocol [44]. Briefly, the recombinant plasmid MSP1E3D1 in pET28a was used to transform *E. coli* BL21 DE3 cells, and transformants were grown in Terrific Broth medium supplemented with 50 μg/mL kanamycin and 2 mm MgSO_4_ at 37 °C. Cells were induced at an OD_600_ of 1.8 with 1 mm IPTG and harvested after 3 h using centrifugation. 20 g cells from 6 L culture were resuspended in 20 mm phosphate buffer pH 7.4 containing 1% Triton X‐100, 1 mm PMSF and 4 μg/mL DNase. Cell lysis was performed using sonication, and debris was removed using centrifugation at 40,000 g for 40 min. The supernatant was applied to a 20 mL HisPrep column pre‐equilibrated with 40 mm phosphate buffer pH 7.4. Three washes of 10 CV each were performed using the following buffer: (a) 40 mM Tris pH 8, 300 mm NaCl, 1% TritonX‐100, (b) 40 mm Tris pH 8, 300 mM NaCl, 20 mm imidazole, 50 mm sodium cholate, (c) 40 mm Tris pH 8, 300 mm NaCl, 50 mm imidazole. The elution was finally performed using 40 mm Tris pH 8, 300 mm NaCl, 200 mm imidazole. Recombinant TEV protease containing a polyhistidine tag was added to the eluate and dialysed overnight against 25 mm Tris pH 7.4, 100 mM NaCl and 0.5 mm EDTA. The cleaved and dialysed protein was further purified through reverse IMAC and concentrated to 10 mg/mL and stored at −80 °C till further use.

### Blue native gel electrophoresis

Blue native polyacrylamide gel electrophoresis (BN‐PAGE) was performed as described previously [45]. Gradient gels (2–12%) were prepared using imidazole–aminocaproic acid buffer and run under native conditions in an imidazole tricine buffer system at 4 °C. Samples were mixed with Coomassie Brilliant Blue G‐250 containing loading dye prior to electrophoresis. Gels were run at 60 V for 2 h, after which the cathode buffer was replaced with a dye‐diluted buffer and electrophoresis was continued at a constant current of 4 mA until the dye front reached the bottom of the gel. DDM‐solubilised ovine heart mitochondria were used as molecular weight markers, with respiratory complexes I (~ 1 MDa), V (~ 600 kDa), III (~ 500 kDa), and IV (~ 200 kDa) serving as reference bands.

### Preparation of lipid stock

*E. coli* polar lipids (Avanti) in chloroform were dried in a glass test tube under a constant stream of argon. Traces of chloroform were removed using overnight desiccation. The dried lipids were resuspended in 50 mm Tris, 200 mm NaCl and 2% CHAPS at a concentration of 20 mg/mL and sonicated at 25 kHz with 2 s on and 6 s off cycle using a microprobe, till the solution became clear. This took an approximate time of 5 min.

### Reconstitution of MprF in nanodisc

PaMprF was purified as above (after Ni‐NTA affinity chromatography) and concentrated to 4 mg/mL. MSP1E3D1 and *E. coli* polar lipids in 2% CHAPS were mixed at a ratio of 1 : 30 and incubated at RT for 30 min before placing on ice. MprF in GDN was added to this mix, such that the ratio of MprF, MSP1E3D1 and lipids was 1 : 4 : 120, and incubated on ice for further 15 min. An equal volume of Biobeads SM2 (BioRad, USA) pre‐equilibrated in Buffer A was added to the mix and allowed to stir gently overnight along with IP_6_ for removal of the CPD tag. After detergent removal, the nanodisc mix was collected and centrifuged at 20,000 g to remove any precipitate before diluting 15 times. This was further purified with StrepTactin column. The eluate was then subjected to size exclusion chromatography on a Superose 6 Increase column. The peak fraction was concentrated to 2.5 mg/mL and used for further experiments.

### Cryo‐EM grid preparation and data acquisition

The top side of Quantifoil Au R1.2/1.3 or Ultrafoil 1.2/1.3 [46] (GDN), Au R 0.6/1.0 (nanodisc) and Au R 1/1 (for cysteine mutants) 300 mesh holey carbon grids were glow discharged at 25 mA for 60 s in air using a PELCO glow discharger (Ted Pella). 3 μL sample was applied on the grid and allowed to stand for 10 s at 16 °C and 100% humidity, followed by blotting for 3–3.5 s with a blot force of 0 and subsequent vitrification in ethane using Vitrobot Mark IV (ThermoFisher Scientific, USA). Data collection was performed with a 300 kV Titan Krios G3i (ThermoFisher Scientific) with a Falcon 3 detector in counting mode at a nominal magnification of 75,000× using the EPU software (ThermoFisher Scientific). The resulting pixel size was 1.07 Å and the image acquisition was performed at a defocus range of −1.5 to −3.0 μm. The dose was measured to be 0.55 e^−^/pixel/s with a total exposure of 60 s and movies were fractionated into 25 frames (the dose for each data set is provided in Table 1).

### Image‐processing, modelling and model refinement

The initial data processing steps were performed in RELION 4.0 [26, 28, 47]. Motion‐correction was performed using RELION, followed by estimation of the contrast transfer function with Gctf [48]. Autopicking was performed using Topaz [49]. For the data set in GDN, a total of 1,239,668 particles were extracted with a box size of 320 pixels, and iterative 2D classification was used to clean up the data, yielding 597,348 particles. An initial model was generated and further 3D refinement with C2 symmetry was performed to obtain a map at a resolution of 4.17 Å. Multiple 3D classification steps were performed to obtain homogeneous particles. In the GDN dataset, classes with well resolved TM helices were chosen for Bayesian polishing with 375 489 particles. These particles were then imported to cryoSPARC [27, 50] for further processing using non‐uniform refinement and local and global CTF refinement to yield a map of ~ 3.2 Å with C1 symmetry applied. Initially, reconstruction and heterogenous refinement was used to separate classes having only one or two soluble domains comprised of 168,060 and 109,157 particles, respectively. The particles/classes with both soluble domains intact were refined using non‐uniform refinement with C1 or C2 symmetry. The final map sharpening was done with RELION PostProcess with automatic B‐factor sharpening or with adhoc B‐factor. Asymmetric refinement and reconstruction of populations containing a single soluble domain was also performed in cryoSPARC similarly, to yield a map at 3.3 Å resolution. The workflow of this data set is provided in Fig. S1.

The nanodisc dataset was collected at the same settings as above (1.07 Å sampling and Falcon 3 in counting mode) and had 3,388 movie stacks that resulted in 1,701,373 particles. After multiple rounds of 2D classification, an initial model was generated using 428,167 particles. 3D classification was used to further remove heterogeneous classes and 138,067 particles were subjected to 3D refinement to yield a map at a resolution of 4.4 Å. After Bayesian polishing, the particles were exported to cryoSPARC for further processing. Non‐uniform refinement in C2 symmetry yielded a map at 3.5 Å, which was sharpened in RELION with automated as well as adhoc B‐factor sharpening. A subset of particles chosen from the initial and heterogenous refinement with no symmetry imposed resulted in a map of 3.6 Å resolution after non‐uniform refinement. The workflow of this data set is provided in Fig. S3.

For the H386C/F389C nanodisc dataset, 892,429 particles were extracted from 2,389 movie stacks, which were curated to 249,116 particles after two rounds of 2D classification. A 3D classification step resulted in 104,195 particles, which was then exported to cryoSPARC and non‐uniform refinement, global and local CTF refinements were performed. The particles were imported back to RELION to perform Bayesian polishing, followed by another round of local and global CTF refinement and non‐uniform refinement in cryoSPARC to yield a map at 3.3 Å resolution with C2 symmetry. The workflow of this data set is provided in Fig. S8.

In the H566C GDN dataset, 808,820 particles were extracted from 2,066 movie stacks, which were curated to 216,425 and 119,348 particles after 2D and 3D classifications, respectively. The downstream processing was similar to the H386C/F389C nanodisc dataset but no symmetry was applied to the 3D refinement and reconstruction, generating a 3.5 Å resolution map. The workflow of this data set is provided in Fig. S9.

For the H566C nanodisc dataset, a total of 1,434,841 particles were extracted from 3,180 micrographs, and iterative 2D classification revealed populations with two dimer interfaces. Particles from both these classes with the different interfaces were selected and processed separately. The major class with 183,514 particles after 3D classification in RELION, was subjected to Ab‐initio reconstruction and heterogenous refinement in cryoSPARC resulting in 131,805 particles that were further processed similar to the other cysteine mutants, resulting in a C2 symmetrised map to a resolution of 3.2 Å. The particles (# 73784) with side views containing well separated synthase domain and a central depression on the periplasmic side were selected and exported to cryoSPARC. Iterative 2D classification followed by initial reconstruction resulted in a low‐resolution map consisting of 14,295 particles. This was followed by homogenous and non‐uniform refinement of the map using C2 symmetry. The workflow of this data set is provided in Fig. S10. 3DFSC plots for all the datasets were generated using the 3dfsc server – https://3dfsc.salk.edu [51].

The model for PaMprF was built in Coot [52] using maps that were sharpened with RELION (auto or adhoc B factor) or deepEMhancer or EMReady [29, 53]. The crystal structure of soluble domain PDB – 4v35 was docked into the map using Chimera and then built in coot [52, 54]. The TM helices were modelled as polyalanine and the assigned sequence was verified with the predicted AlphaFold model [55]. The lipid densities were identified by calculating FSC weighted difference (fofc) maps using servalcat [34]. Refmac5/servalcat was used to generate the other monomer based on C2 symmetry. Model refinement was performed in real space refinement with Phenix or in reciprocal space with Refmac [56, 57, 58, 59]. The figures were made with pymol, Chimera or chimerax [30, 32, 54]. The cavity analysis was performed using KVFinder web server [60] and the topology of PaMprF was made with PDBsum [61]. The interface analysis of PaMprF and RtMprF were performed with PDBePISA server [62]. Sequence analysis was performed with ClustalX and JalView [63, 64] and JPred for secondary structure prediction [65, 66] and analysis of conserved residues with consurf server [40, 67].

### Cysteine cross‐linking

The wild type and mutant membranes were thawed and diluted to a concentration of 1 mg/mL, using buffer A with or without β‐mercaptoethanol. The membranes were treated with either DMSO (control) or 2 mm BMOE (Cat# 22323; ThermoFisher‐Pierce) or 100 μm CuPt and incubated for 30 min at room temperature. Samples were denatured using non‐reducing SDS loading dye (or 100mm β‐mercaptoethanol was added to the samples prior to cross‐linker addition and were loaded separately, labelled as reducing), and SDS‐PAGE gels were run, followed by transfer to a PVDF membrane. The membrane was probed using HRP conjugated antibody against Strep Tag and developed using ECL substrate.

Cross‐linking of purified PaMprF H386C/F389C was performed by adding ~ 100 μm BMOE to the membrane prior to detergent solubilisation, followed by purification and nanodisc reconstitution as described for the PaMprF WT. Size‐exclusion chromatography revealed two major peaks at 11.3 and 13.2 mL, respectively, which were subsequently analysed by SDS‐PAGE and BN‐PAGE. The sample corresponding to the peak at 13.2 mL was used for cryo‐EM. Alternatively, ~ 100 μm CuPt was added to purified PaMprF H386C/F389C in GDN for SDS PAGE analysis. For the H566C mutant purification, no cross‐linker was added in the membranes or during purification but only prior to the gel run. Instead, samples were analysed directly under detergent and nanodisc conditions to evaluate the proximity of the two H566C residues within the dimeric complex in the two conditions. All these samples were similarly analysed using SDS‐PAGE and BN‐PAGE.

## Author contributions

SJ performed all the experiments including sample preparation, cryo‐EM data collection, image‐processing, model‐building and wrote the manuscript. KRV was involved in image‐processing and model‐building, writing of the manuscript and supervision of the project.

## Conflicts of interest

The authors declare no conflicts of interest.

## Supporting information


**Fig. S1.** Cryo‐EM analysis of PaMprF purified in GDN detergent.
**Fig. S2.** Cryo‐EM map of PaMprF expressed and purified with a different construct.
**Fig. S3.** Cryo‐EM analysis of PaMprF purified in nanodisc.
**Fig. S4.** Select cryo‐EM densities of PaMprF from the GDN and the nanodisc reconstructions.
**Fig. S5.** Structural description of PaMprF.
**Fig. S6.** Effect of detergents on the oligomeric state of PaMprF.
**Fig. S7.** Biochemical analysis of PaMprF WT and the mutant enzymes.
**Fig. S8.** Cryo‐EM analysis of PaMprF H386C/F389C in nanodisc with BMOE added to the membrane, prior to detergent solubilisation.
**Fig. S9.** Cryo‐EM analysis of PaMprF H566C in GDN.
**Fig. S10.** Cryo‐EM analysis of PaMprF H566C in nanodisc.
**Fig. S11.** Cryo‐EM density of select regions of PaMprF mutants.
**Fig. S12.** Unusual and less populated classes in PaMprF datasets.
**Fig. S13.** Sequence alignment of select MprF homologues.

## Data Availability

The cryo‐EM maps and the coordinates are deposited in the EMDB and PDB, respectively, with the following accession codes. PaMprF in GDN, C2 symmetry—EMD‐61238 and PDB 9J8Q, PaMprF in GDN, C1 symmetry—EMD‐61239 and PDB 9J8R, PaMprF in nanodisc—EMD‐61240 and PDB 9J8S, PaMprF H386C/F398C in nanodisc—EMD‐65648 and PDB 9W50, PaMprF H566C in nanodisc—EMD‐65649 and PDB 9W51.
