# Supplementary material for: Cryo‐EM structures of multiple‐peptide resistance factor (MprF) from Pseudomonas aeruginosa
Source: FEBS J. 2026 May 22;293(17):5318–39. doi: 10.1111/febs.70519 (PMC13534887; doi:10.1111/febs.70519)
Supplement: Supplementary file 1 [file FEBS-293-5318-s001.pdf]

Supplementary information:

Cryo-EM structures of multiple-peptide resistance factor (MprF) from *Pseudomonas aeruginosa*

Shaileshanand Jha and Kutti R. Vinothkumar *

National Centre for Biological Sciences, Tata Institute for Fundamental Research, Bellary Road, Bangalore 560065.

*Address for correspondence: vkumar@ncbs.res.in

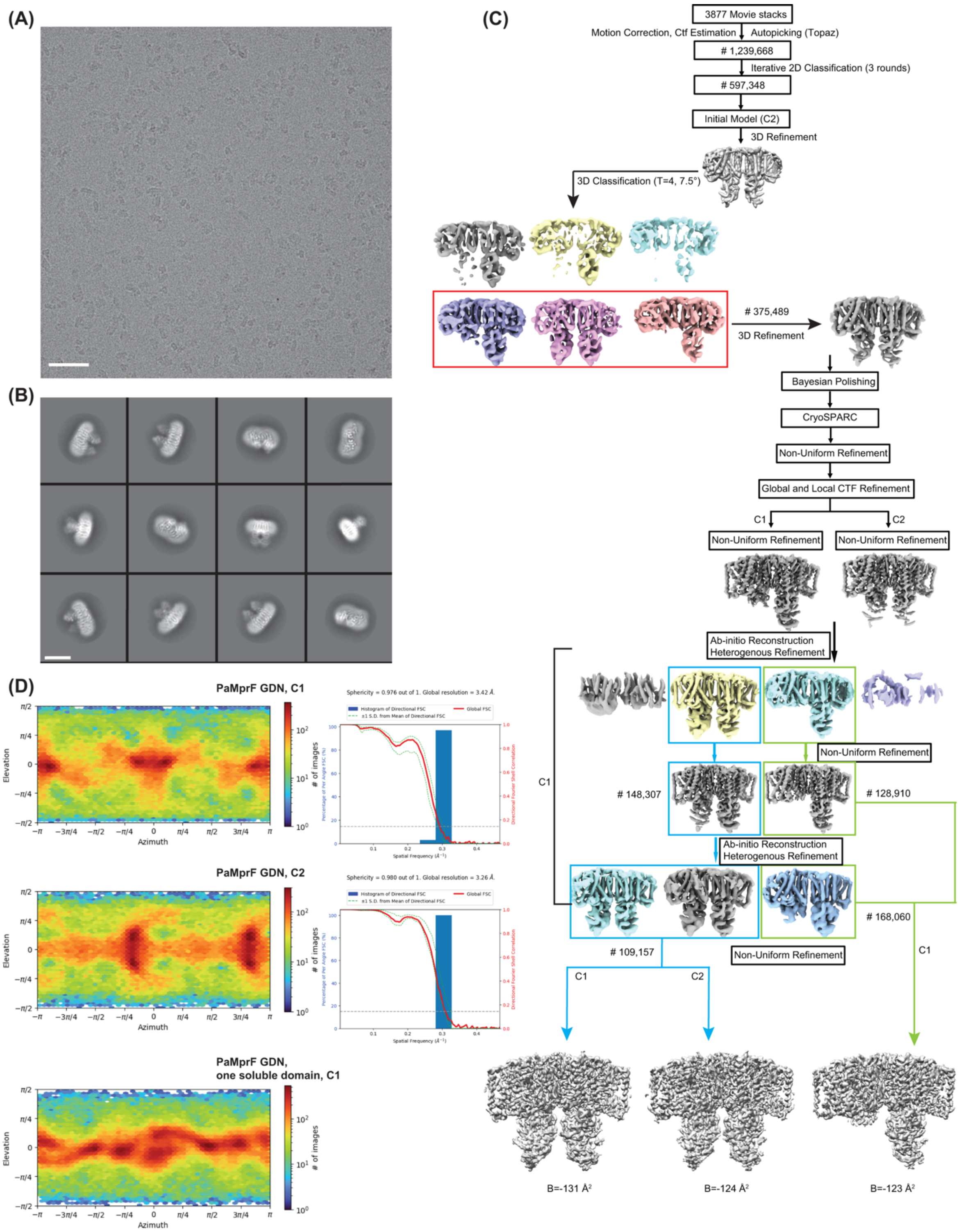
(A)
(B)
(C)
(D)
3877 Movie stacks
Motion Correction, Ctf Estimation
Autopicking (Topaz)
1,239,668
Iterative 2D Classification (3 rounds)
597,348
Initial Model (C2)
3D Refinement
3D Classification (T=4, 7.5°)
375,489
3D Refinement
Bayesian Polishing
CryoSPARC
Non-Uniform Refinement
Global and Local CTF Refinement
C1
C2
Non-Uniform Refinement
Non-Uniform Refinement
Ab-initio Reconstruction
Heterogenous Refinement
C1
Non-Uniform Refinement
148,307
128,910
Ab-initio Reconstruction
Heterogenous Refinement
168,060
109,157
Non-Uniform Refinement
C1
C1
C2
B=-131 Å²
B=-124 Å²
B=-123 Å²
PaMprF GDN, C1
Sphericity = 0.976 out of 1. Global resolution = 3.42 Å.
PaMprF GDN, C2
Sphericity = 0.980 out of 1. Global resolution = 3.26 Å.
PaMprF GDN,
one soluble domain, C1
Elevation
Azimuth
# of images

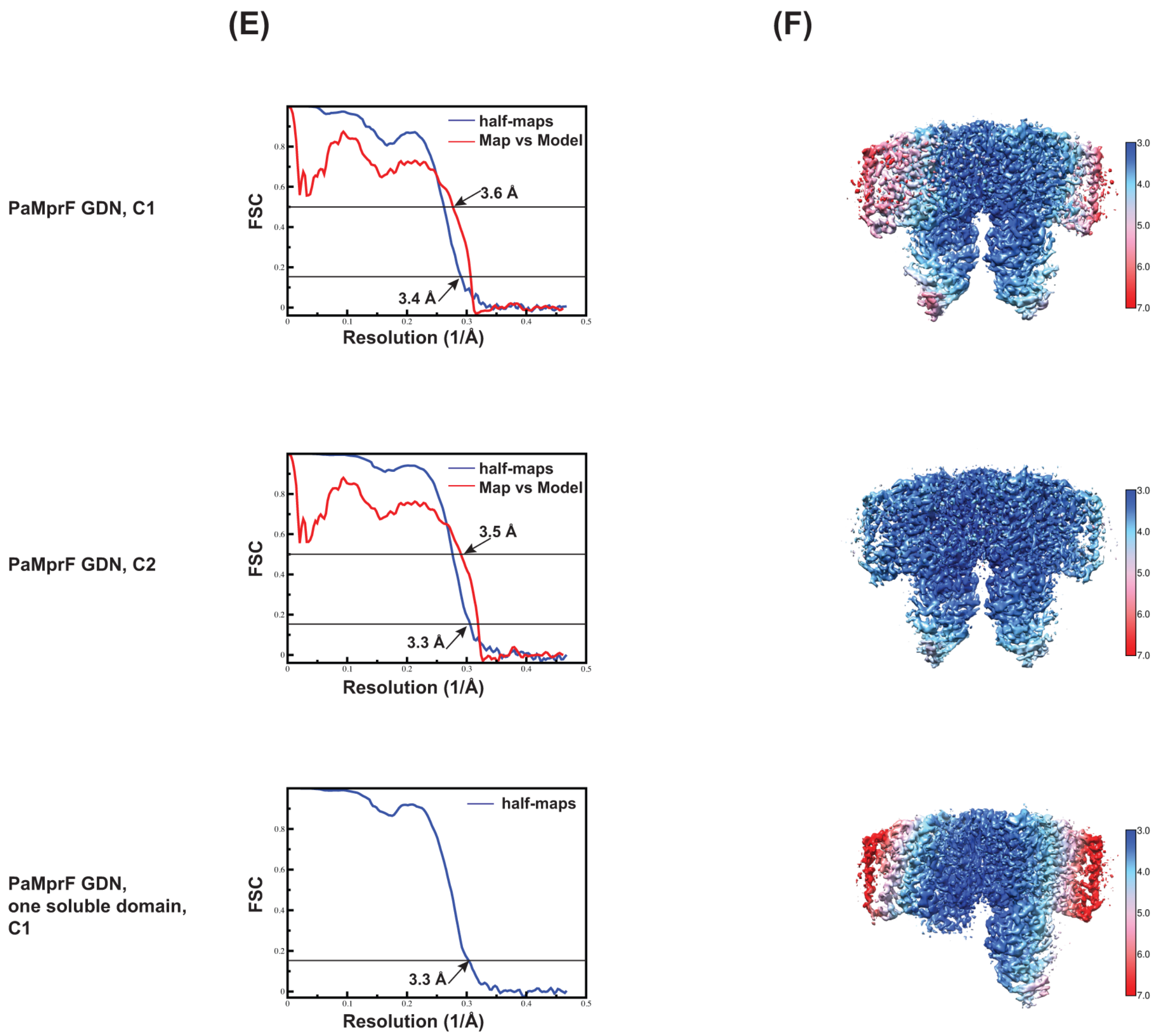

Figure S1: Cryo-EM analysis of PaMprF purified in GDN detergent. (A) Representative micrograph showing MprF particles. Scale bar is 500 Å. **(B)** Representative 2D class averages with a box size of 320 pixels or 342.4 Å. Scale bar is 100 Å. **(C)** Cryo-EM data processing workflow for PaMprF in GDN. Both RELION and cryoSPARC were used for image processing. The final sharpened maps and the B-factor value used for sharpening are given. (**D)** The orientation distribution plots of all the three reconstructions of PaMprF obtained from cryoSPARC represented as heatmaps and the 3DFSC of plots of PaMprF in GDN with C1 and C2 symmetry imposed are shown alongside. **(E)** Fourier Shell Correlation curves of the cryo-EM maps of PaMprF (GDN) with C1 and C2 refinement of the particles with both soluble domains ordered and the particles with only one soluble domain. The resolution is estimated from the FSCs by comparing the half-maps at 0.143 (blue colour) and the map vs model at 0.5 (red colour). Model building and refinement was not performed for the map obtained with only one ordered soluble domain. **(F)** Local Resolution of PaMprF cryo-EM maps as estimated by RELION. Colour key is given on the right with blue and red representing 3 Å and 7 Å respectively.

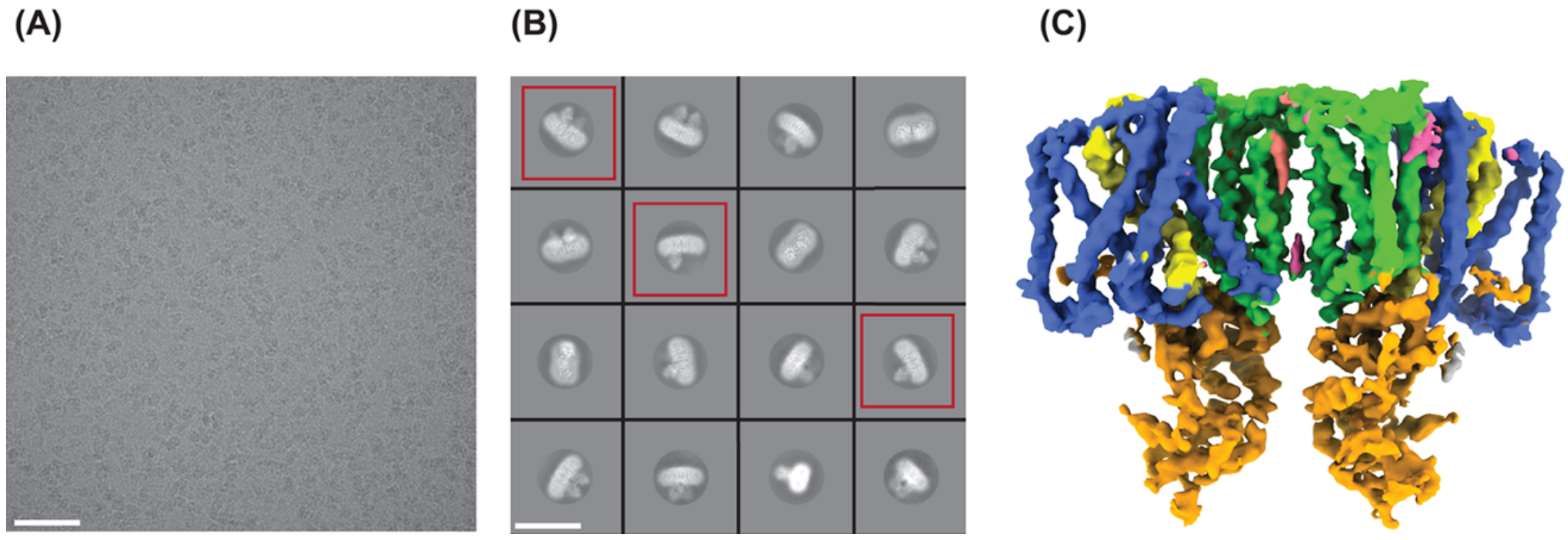

Figure S2. Cryo-EM map of PaMprF expressed and purified with a different construct. PaMprF was expressed and purified with a PreScission cleavage site and a poly-histidine tag at its C-terminus instead of the CPD tag. **(A and B)**, Representative micrograph and reference-free 2D class averages of PaMprF with a human rhinovirus (HRV) 3C cleavage site. The scale bar in the micrograph is 500 Å and the box size of the 2D classes are 320 pixels (sampled at 1.07 Å/pixel). Scale bar is 100 Å. Few classes marked in red square show the populations with only a single soluble domain ordered, a common feature in both the constructs of PaMprF (with CPD and without CPD). **(C)** Cryo-EM map of PaMprF purified in GDN using a HRV 3C cleavage site and the tag was cleaved before the sample prep for cryo-EM. The resolution of the map is ~ 4Å with C2 symmetry applied and coloured as in figure 1. Figure was made with ChimeraX [30].

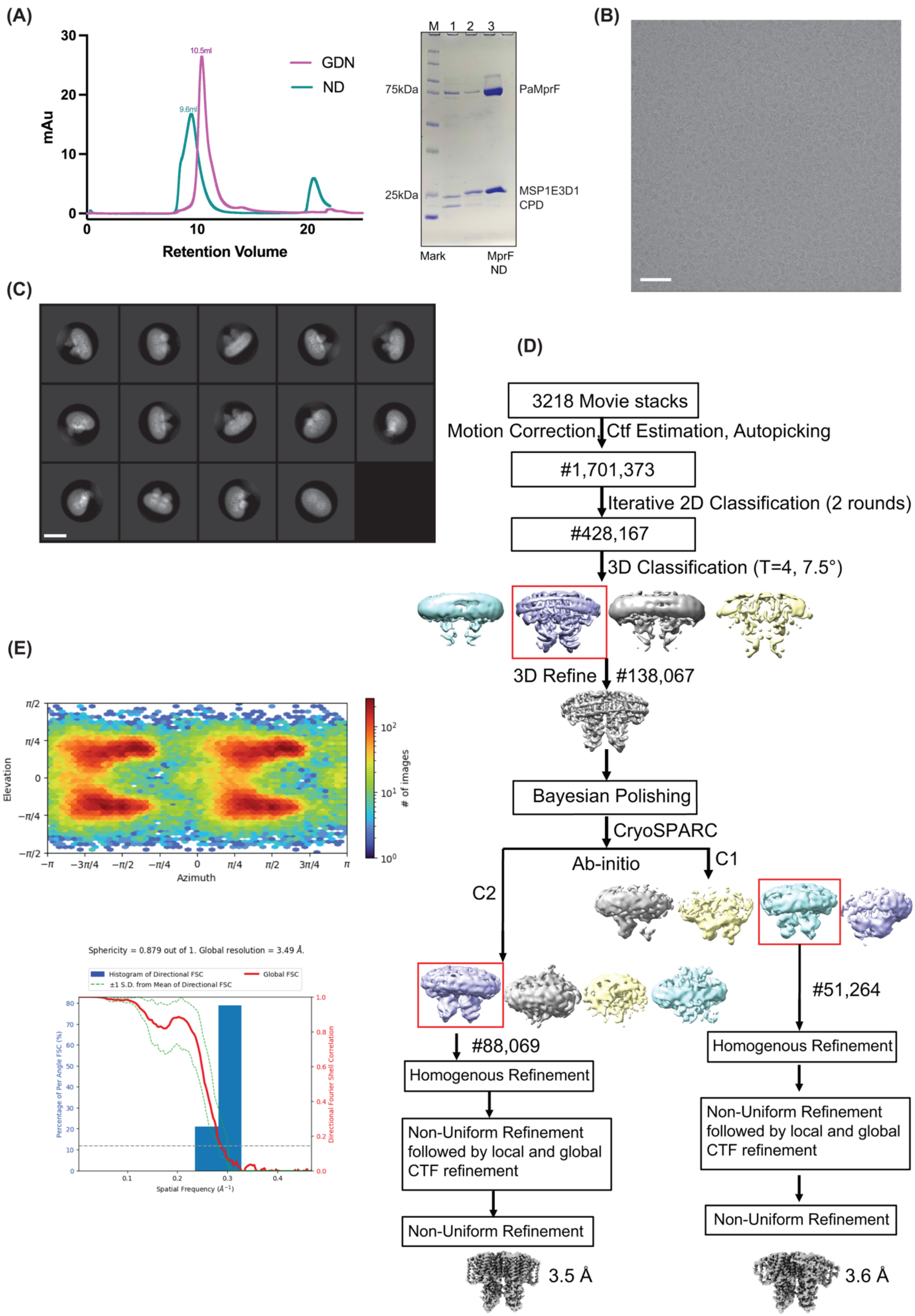

(A)
30
20
10
0
mAu
10.5ml
9.6ml
GDN
ND
0
10
20
Retention Volume
M 1 2 3
75kDa
25kDa
PaMprF
MSP1E3D1
CPD
Mark
MprF
ND
(B)
(C)
(D)
3218 Movie stacks
Motion Correction, Ctf Estimation, Autopicking
#1,701,373
Iterative 2D Classification (2 rounds)
#428,167
3D Classification (T=4, 7.5°)
3D Refine
#138,067
Bayesian Polishing
CryoSPARC
Ab-initio
C1
C2
#51,264
#88,069
Homogenous Refinement
Non-Uniform Refinement followed by local and global CTF refinement
Non-Uniform Refinement
3.5 Å
3.6 Å
(E)
Elevation
Azimuth
# of images
Sphericity = 0.879 out of 1. Global resolution = 3.49 Å.
Histogram of Directional FSC
Global FSC
±1 S.D. from Mean of Directional FSC
Percentage of Per Angle FSC (%)
Directional Fourier Shell Correlation
Spatial Frequency (Å⁻¹)

(F)

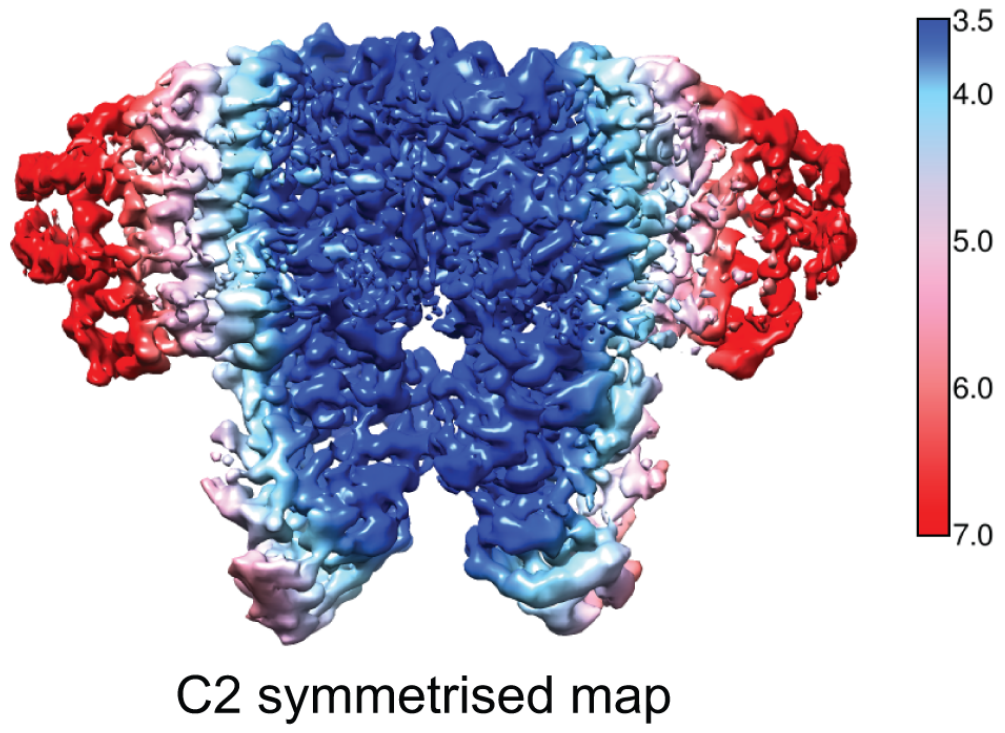

(G)

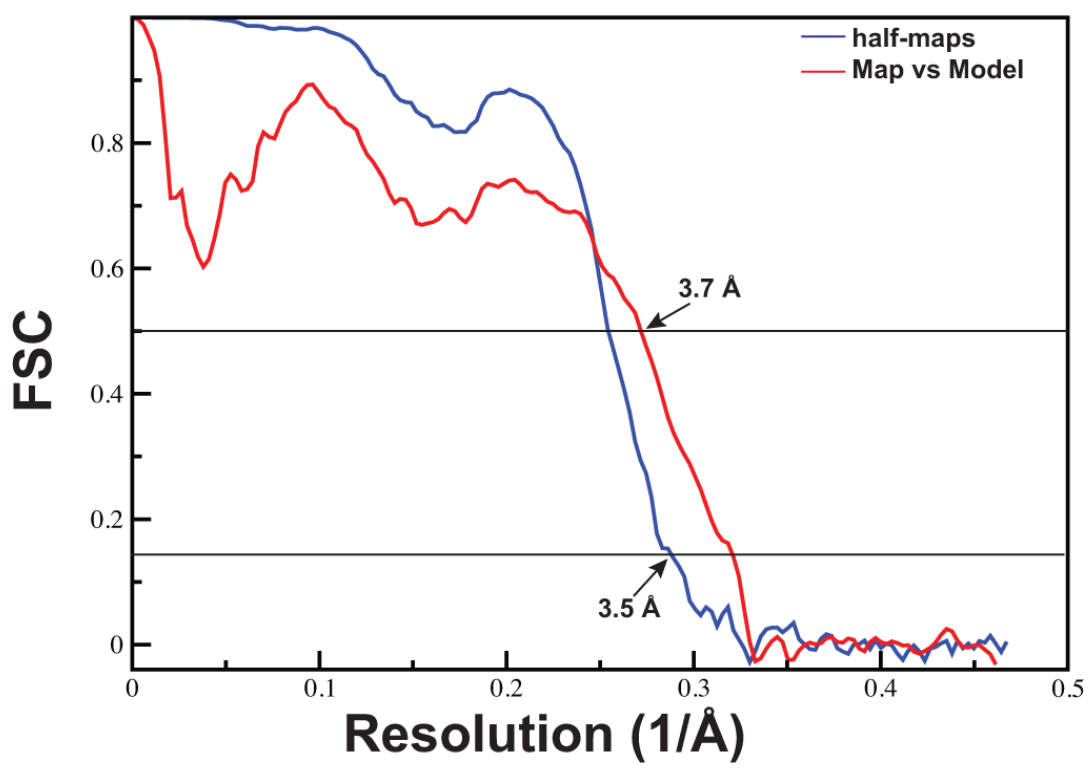

Figure S3: Cryo-EM analysis of PaMprF purified in nanodisc. (A) Reconstitution of MprF in nanodisc. The size exclusion chromatography profile and the gel showing the enzyme and nanodisc protein indicative of successful reconstitution. When compared to MprF in GDN, the nanodisc is profile is slightly shifted and broad. A SDS PAGE gel showing the reconstitution of PaMprF in nanodisc (n=>3). Lane M is marker, Lane 1 is PaMprF after Ni-NTA and reconstituted with MSP1E3D1 and addition of IP_6 to remove the CPD tag, Lane 2- eluate from Strep affinity column to remove the CPD and the his-tag and lane 3 – is the fraction after gel filtration and concentration showing both PaMprF and MSP1E3D1. **(B)** A representative micrograph of PaMprF in nanodisc showing the particles. Scale bar is 500 Å. **(C)** Representative 2D class averages of MprF in nanodisc with a box size of 320 pixels or 342.4 Å. Scale bar is 100 Å. **(D)** Image processing workflow of MprF in nanodisc. **(E)** Orientation distribution plot of the PaMprF obtained from cryoSPARC represented as a heatmap. The 3D FSC plot of PaMprF in nanodisc with C2 symmetry imposed is shown below, indicating some anisotropy. **(F)** Local Resolution of PaMprF (C2 symmetry) as estimated by RELION. Colour key shows the core of the enzyme is around 3.5 Å with the periphery and the nanodisc belt at lower resolution. **(G)** Fourier Shell Correlation curve of PaMprF (nanodisc) with C2 symmetry. The resolution is estimated from the FSCs by comparing the half-maps at 0.143 (blue colour) and the map vs model at 0.5 (red colour) reveals 3.5 and 3.7 Å respectively.

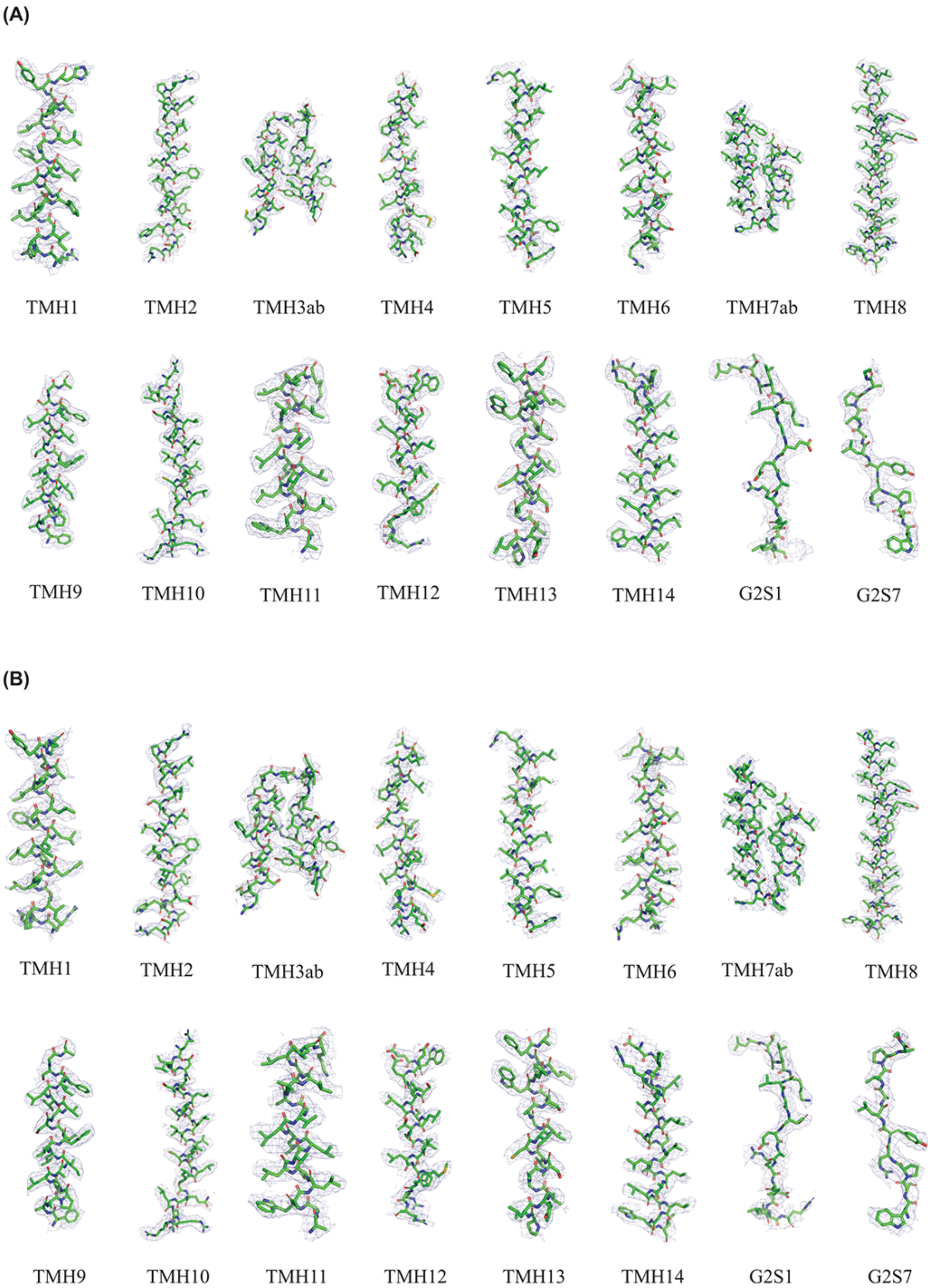

(A)
TMH1
TMH2
TMH3ab
TMH4
TMH5
TMH6
TMH7ab
TMH8
TMH9
TMH10
TMH11
TMH12
TMH13
TMH14
G2S1
G2S7
(B)
TMH1
TMH2
TMH3ab
TMH4
TMH5
TMH6
TMH7ab
TMH8
TMH9
TMH10
TMH11
TMH12
TMH13
TMH14
G2S1
G2S7

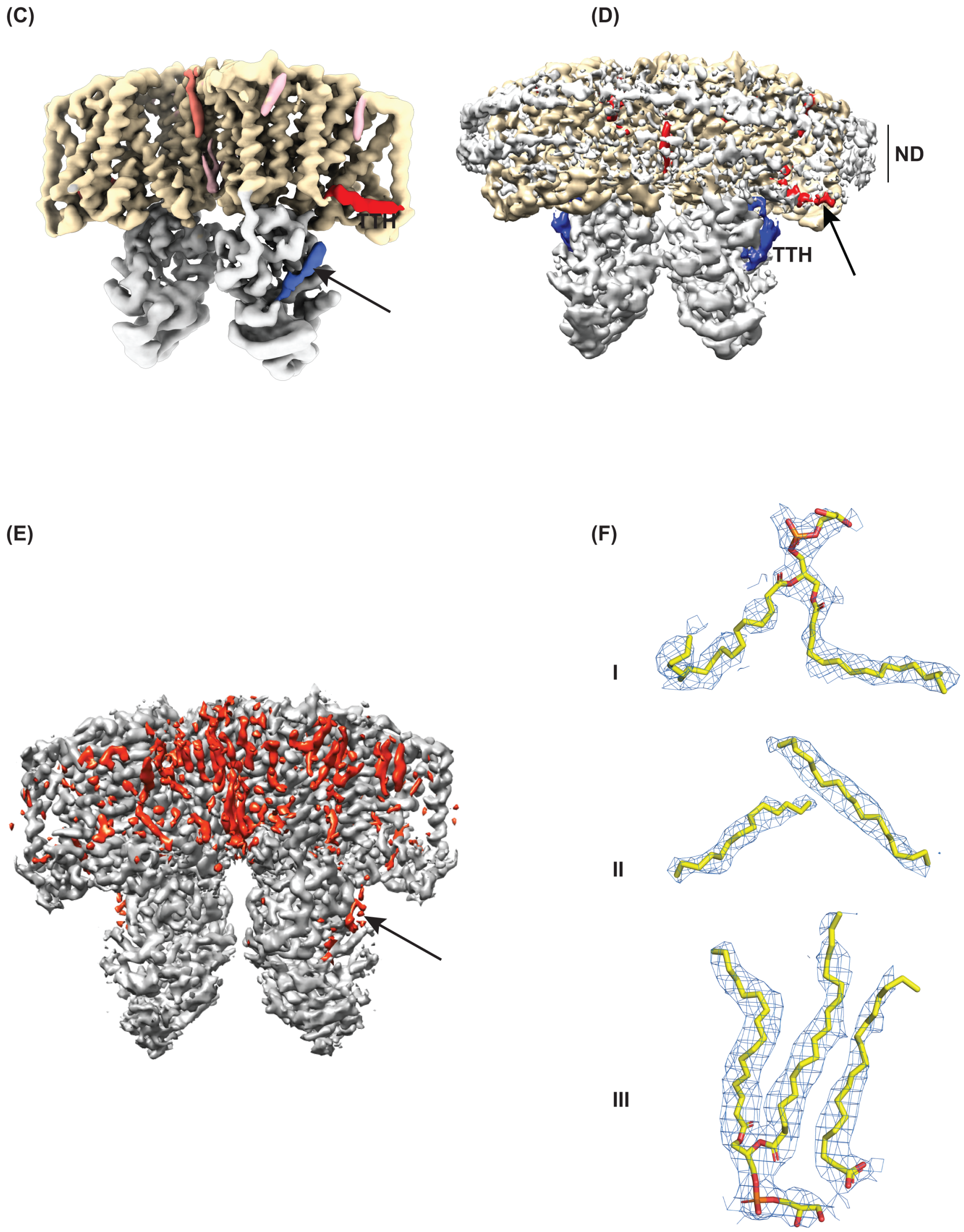

Figure S4: Select cryo-EM densities of PaMprF from the GDN and the nanodisc reconstructions. Fit of the model to the cryo-EM density of the various TM helices and β-sheets into the GDN **(A)** and nanodisc **(B)** maps. The model is shown in stick representation and the carbon atoms in green colour is encased in a blue mesh of the final sharpened cryo-EM map contoured at 4 σ. **(C)** Unmodelled tubular density in the cryo-EM map of PaMprF GDN. An unsharpened map of PaMprF (GDN) containing both the soluble domains reconstructed using C2 symmetry. The TM domain, soluble domain and the TTH are coloured wheat, grey and red. The additional unmodelled density in the soluble domain is coloured in blue and marked with black arrow. **(D)** Unmodelled density in the cryo-EM map of PaMprF nanodisc. The cryo-EM map of PaMprF in nanodisc sharpened with a B factor of -65 $Å^2$ is shown here with the TM and soluble domain coloured in wheat and grey. The density for TTH is shown in blue and the arrow marking the red density, which is currently unmodelled occupies a similar place as the TTH in GDN shown in panel C is most likely a lipid molecule. The nanodisc belt (grey) in the cryo-EM map can be clearly seen in this map. **(E)** Cryo-EM (sharpened) map of PaMprF, showing unmodelled densities

in red and the modelled protein density in grey. The lipid-like densities are clearly seen in the membrane domain and the fragmented density marked with arrow is the unmodelled density in the soluble domain (same as in panel C). The figure was made in Chimera with colour zone and split map option using the PaMprF model without any lipids included. To show different regions and densities in the maps, the maps in panels D-E are at different scale and threshold. **(F)** Density of select lipid molecules (as shown in figure 5A) at 5 σ and carved 2 Å around the atoms. The position of the lipids as I, II and III are as shown in main figure 5A. PyMOL [32] was used to make figures in panels A, B and F.

(A)

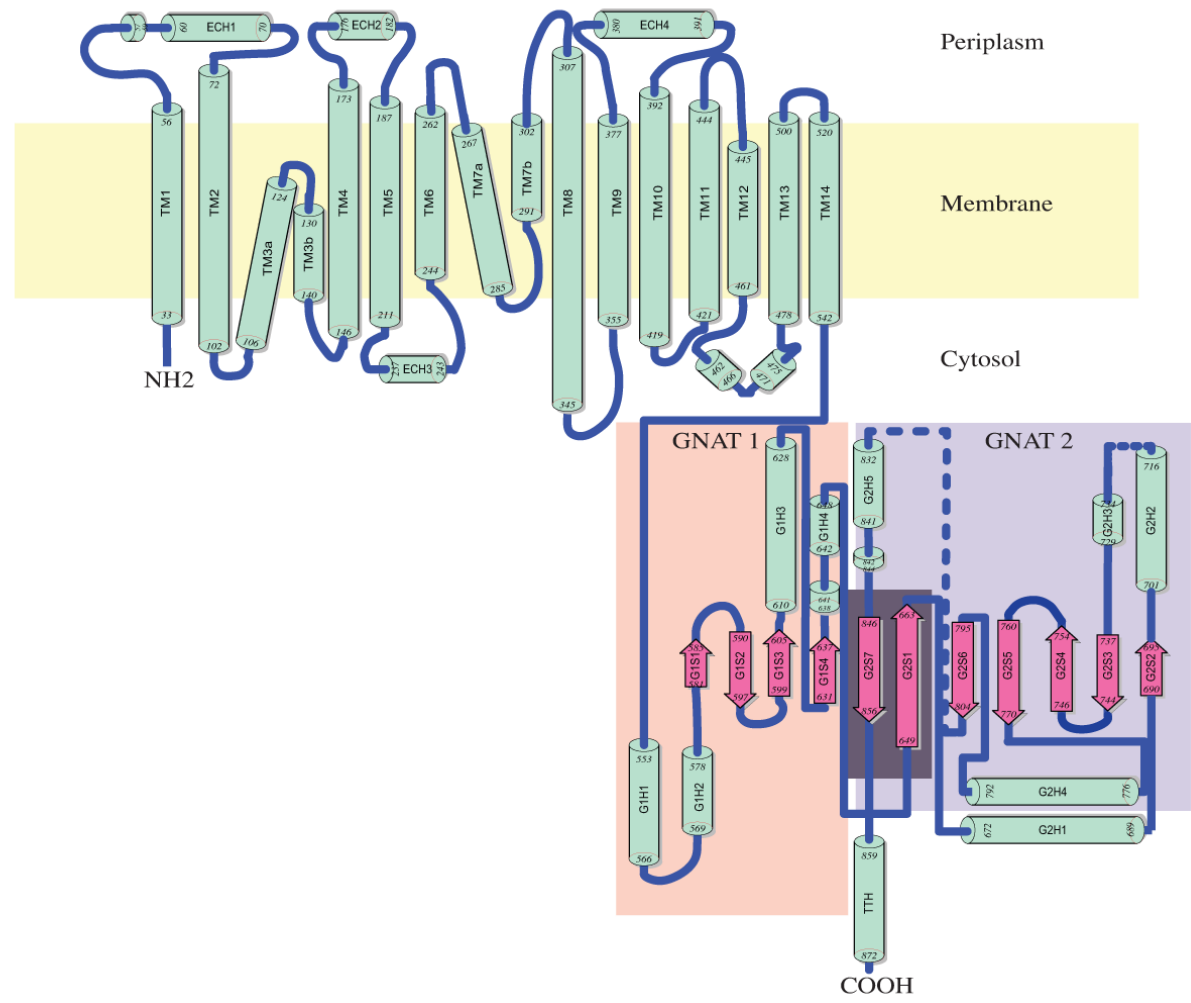

(B)

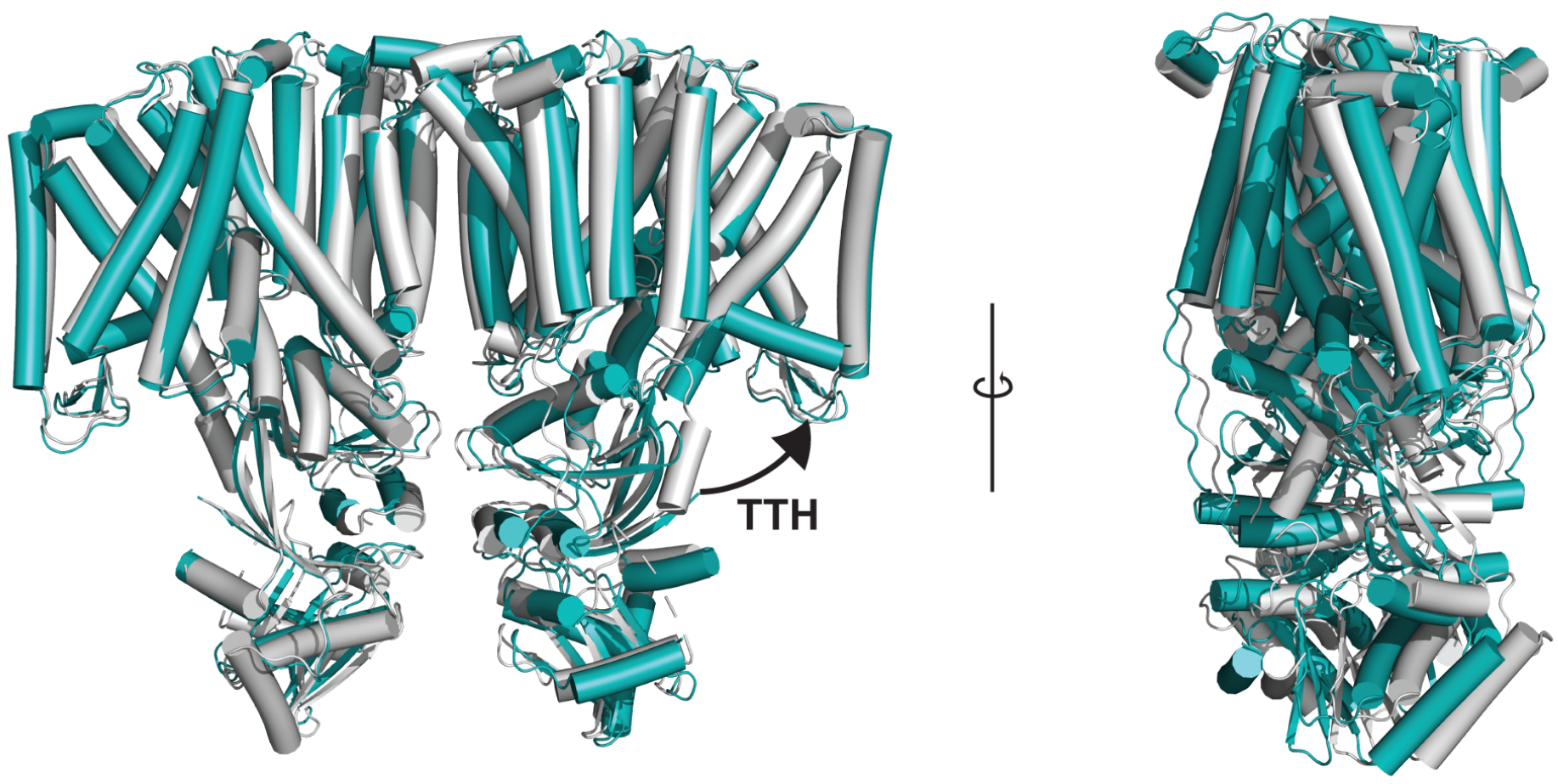

(C)

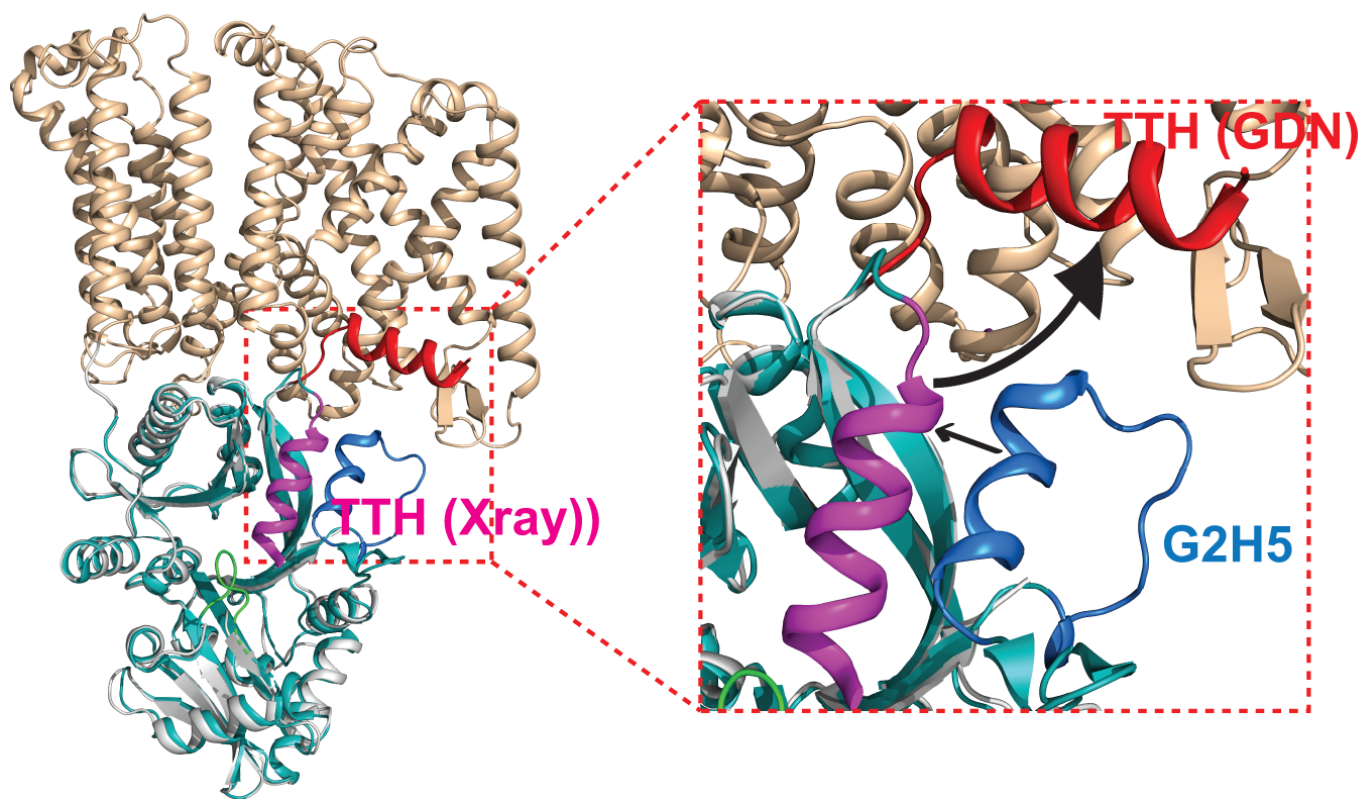

Figure S5: Structural description of PaMprF. (A) Topology diagram of PaMprF. The TM helices (membrane domain) are at the N terminus of the polypeptide chain. The secondary structural elements – α-helices and β-sheets are coloured in green and magenta respectively. The secondary structures are scaled to the length and the first and terminal residues are numbered. The membrane region is shaded in yellow and the TM helices are numbered as

TMH1-14. The cytosolic domain constitutes two GNAT domains 1 and 2 (shaded in colour of salmon and purple). The helices and sheets of the cytosolic domain are named corresponding to the GNAT domain 1 or 2 from N to C-termini. For example, G2S1 corresponds to the second GNAT fold and first β-strand. The strands 5 and 11 are shared by both the GNAT domains. The topology diagram was generated with PDBsum server [61]. **(B)** Comparison of the model of PaMprF purified in GDN (teal) and nanodisc (grey). The tilt of one of the monomers in the nanodisc model is apparent. **(C)** Comparison of models from the full-length PaMprF (GDN) and the crystal structure of the synthase domain. An overlay of the monomer model of PaMprF (GDN) and the soluble domain of the X-ray structure of PaMprF (4v35). The region missing in the cryo-EM PaMprF map and the model belonging to the helix 5 of second GNAT fold (G2H5) is coloured blue. The TTH in the X-ray structure of the soluble domain is coloured magenta, which is similar to the position of TTH in the nanodisc model. The inset on the right indicates the possible movement of G2H5 towards the membrane as shown here in the GDN model (helix in red). Figures in panel B and C were made with PyMOL [32].

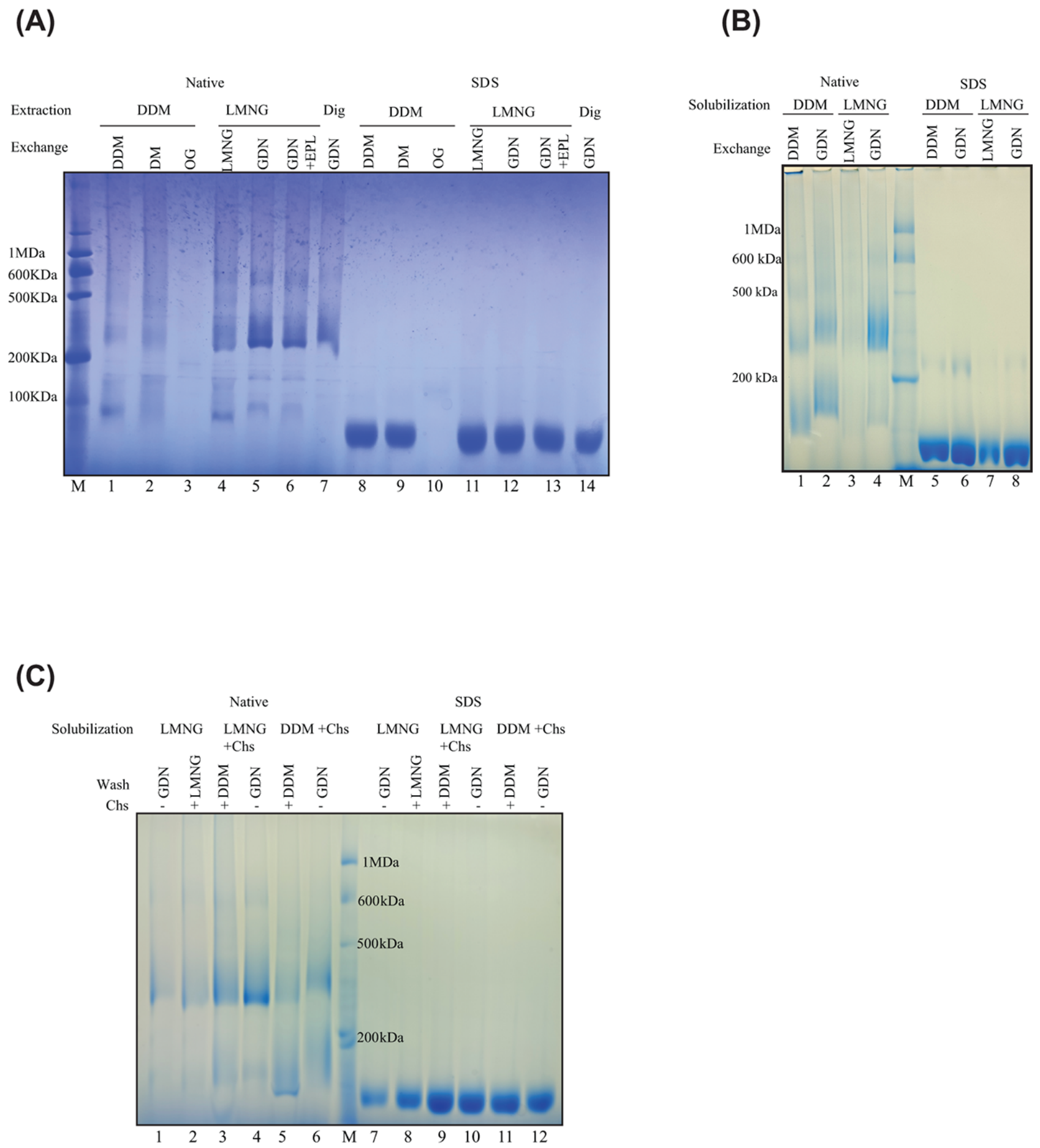

Figure S6: Effect of detergents on the oligomeric state of PaMprF. **(A)** Detergent screen of PaMprF assessed by BN-PAGE showing native samples immediately after the marker lane (M) followed by SDS treated samples as control, where the oligomers dissociate into monomers. In this experiment, solubilisation was performed either with DDM (lanes 1-3), LMNG (lanes 4-6) or digitonin (labelled as Dig) (lane 7) and the detergent used for purification was different (exchanged on the column). Detergents used for purification (from lane 1-7) include DDM, DM, OG, LMNG, GDN, GDN with *E. coli* polar lipids (EPL), and GDN, respectively. Lanes 8-14 are equivalent to lanes 1-7 but treated with 1% SDS showing monomeric band except for OG, where no clear band was observed. 10 µg of purified protein was loaded in each lane. This is a representative BN-PAGE gel of detergent screen that was performed only once for demonstration of the effect of detergents. **(B)** BN-PAGE showing the comparison of DDM or LMNG extraction on PaMprF. Lanes 1-4 and 5-8 are native and SDS treated samples, respectively. Note the difference in the profile of lane 2 and 4 indicating the dissociation of DDM solubilised PaMprF, even when exchanged to GDN. Approximately 15 µg PaMprF was loaded in each lane. This experiment was performed twice. **(C)** BN-PAGE showing the effect of cholesterol hemi succinate (CHS) during extraction and purification on the stability of PaMprF. On-bead cleavage was performed using equal volumes of IP_6 buffer and 20 µl was loaded in each lane. The native samples in lanes 1-6 and their corresponding SDS treated samples (lanes 7-12) indicate the varying stability and yield in different conditions tested. Note the band intensity of LMNG CHS solubilised PaMprF in lane 10, compared to the one without CHS in lane 7. The effect of CHS was analysed only once, and since no major improvement was observed it was not pursued further. The marker in all the 3 BN-PAGE gels shown here is solubilised mitochondrial fraction in DDM.

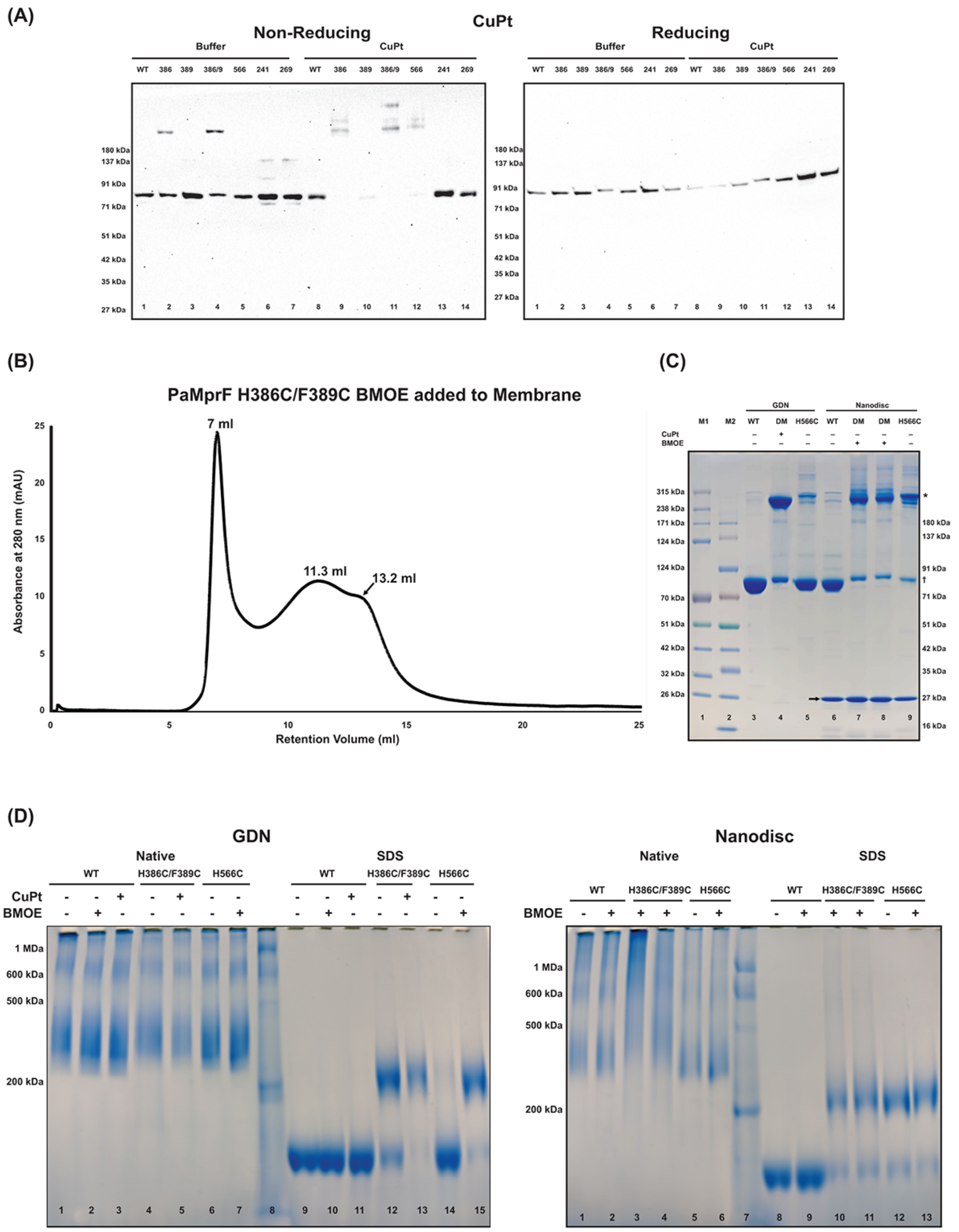

Figure S7: Biochemical analysis of PaMprF and the mutant enzymes. Crosslinking experiment of *E. coli* membranes expressing PaMprF and purified MprF with SDS and BN-PAGE gels. **(A)** The western blots showing cross-linking of PaMprF with copper phenonthroline (CuPt). Lanes 1-7 correspond to membrane samples of WT, H386C, F389C, H386C/F389C, H566C, L241C and A269C with buffer control. Lanes 8-14 correspond to the same samples with CuPt added. The samples on the left and right blot are in non-reducing (n=3) and reducing conditions (n=1, it was performed only once to make sure that the higher molecular bands were formed via cysteines and are specific) respectively. Lane 4 in the non-reducing blot shows that the H386/F389 mutated to cysteine forms a higher molecular weight band even without the addition of the CuPt, indicating the proximity of the residues in the membrane (in lane 2, H386C mutation also shows higher

molecular weight band without addition of CuPt). 10 μg of total membrane protein was loaded in each lane. The inability of crosslinking by CuPt under reducing conditions indicate that these oligomers formed are mediated by disulphide bonds. Note that no crosslinking is observed in lanes 13-14 corresponding to cysteine mutants at the rhizobium like interface of PaMprF. There are additional bands in these lanes, which have also been observed in WT, also observed in reducing conditions and are unlikely to be disulphide linked as their intensity does not increase upon the addition of crosslinker. **(B)** Size exclusion chromatogram of PaMprF H386C/F389C in nanodisc on a Superose 6 column (n=1, the mutant enzyme was purified once for cryo-EM analysis). The aggregates appear at the void volume of ~7 ml and two peaks corresponding to 11.3 and 13.2 ml were observed. **(C)** SDS PAGE of purified PaMprF cysteine mutants (n=1, this particular gel with all samples was run once). Lanes 1 and 2 correspond to protein markers from Puregene, Cat# PG-PMT0782 and #PG-PMT2962 respectively. The corresponding molecular weights of ladders in lanes 1 and 2 are mentioned on the left and right side of the gel respectively. Lanes 3 and 5 are WT and H566C in GDN and lanes 6 and 9 correspond to WT and H566C in nanodisc, respectively. Lane 4 is H386C/F389C (labelled as DM for double mutant) in GDN in the presence of CuPt and lanes 7 and 8 are the two peaks from gel filtration chromatography of the double mutant in nanodisc where membrane was pre-incubated with BMOE. The symbols † and * denote the monomeric and dimeric species of PaMprF. The MSP1E3D1 protein is labelled with an arrow. 8 μg protein is loaded in each lane. **(D)** BN-PAGE of purified PaMprF cysteine mutants (n=1, this particular gel with all samples was run once). PaMprF in GDN resolved in the BN-PAGE is shown on the left. Lanes 1-3 are WT, WT with BMOE and CuPt, respectively. Lanes 4 and 5 are PaMprF H386C/F389C double mutant in the absence and presence of CuPt. Lanes 6 and 7 correspond to PaMprF H566C in the absence and presence of BMOE. Lane 8 is marker and lanes 9-15 are samples corresponding to lanes 1-7 but with the addition of 1% SDS. When treated with SDS and run on BN-PAGE, a higher band along with the monomeric band is observed (example lanes 12, 13 and 15) in the mutants. Note that there is no significant shift in electrophoretic mobility for any of the mutants (with or without crosslinkers) when compared to the WT indicating the oligomer in SDS PAGE and BN-PAGE is indeed a dimer as observed in the WT structure. The gel on the right was loaded with PaMprF in nanodisc. Lanes 1 and 2 are WT, 3 and 4 are H386C/F389C double mutant and lanes 5 and 6 are H566C. BMOE was added to samples after purification in lanes 2 and 6 corresponding to WT and H566C. The H386C/F389C double mutant samples was purified from membrane pre-incubated with ~100 μM BMOE. The two samples correspond to the two peaks obtained in the gel filtration chromatography using Superose 6 column at 11.3 ml (lane 3) and 13.2 ml (lane 4) respectively (from panel B). Lanes 12 and 13 in the ND gel indicate that the H566C mutants in nanodisc readily crosslinks even in the absence of crosslinker but not so in the GDN micelle (lane 14, in left panel), correlating with the observations from the structures of WT in GDN and nanodisc. Approximately 15 μg of protein is loaded in each lane.

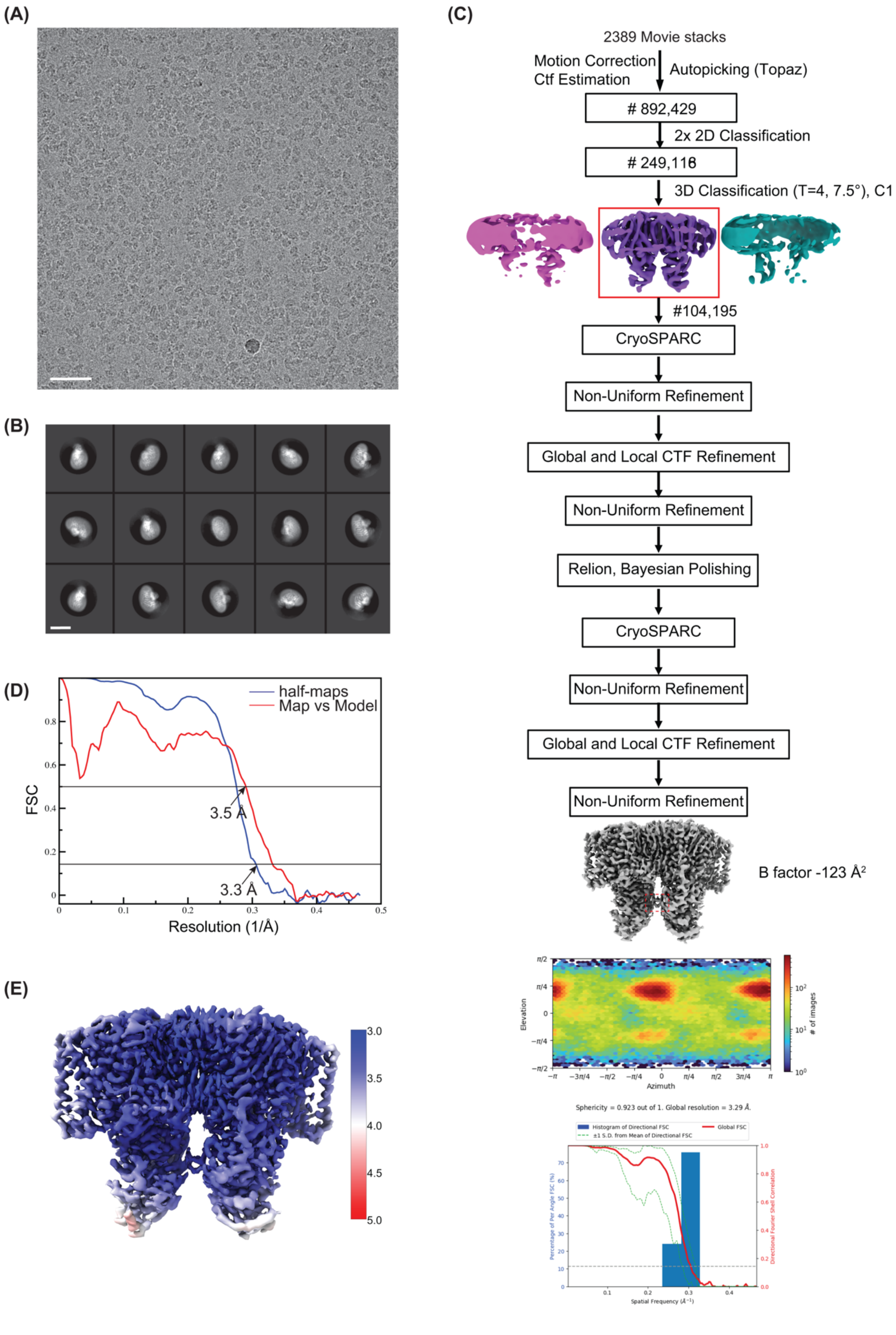

Figure S8: Cryo-EM analysis of PaMprF H386C/F389C in nanodisc with BMOE added to the membrane, prior to detergent solubilisation. (A) A representative micrograph showing the H386C/F389C mutant reconstituted in nanodisc in ice. Scale bar is 500 Å. **(B)**

Representative 2D class averages of PaMprF H386C/F389C in nanodisc with a box size of 320 pixels or 342.4 Å. Scale bar is 100 Å. **(C)** Image processing workflow of PaMprF H386C/F389C in nanodisc using both RELION and cryoSPARC. The orientation distribution plot of the final map PaMprF H386C/F389C in nanodisc obtained from cryoSPARC represented as a heatmap. The 3D FSC plot of PaMprF H386C/F389C in nanodisc with C2 symmetry imposed is shown below. **(D)** Fourier Shell Correlation curve of PaMprF H386C/F389C in nanodisc. The resolution is estimated from the FSCs by comparing the half-maps at 0.143 is ~3.3 Å (blue color) and the map vs model FSC at 0.5 gives a resolution of 3.5 Å (red color). **(E)** Local Resolution of PaMprF H386C/F389C in nanodisc as estimated with RELION. Colour key on the right shows the core of the enzyme is around 3 Å with the periphery at lower resolution. The figure was made with Chimera [54].

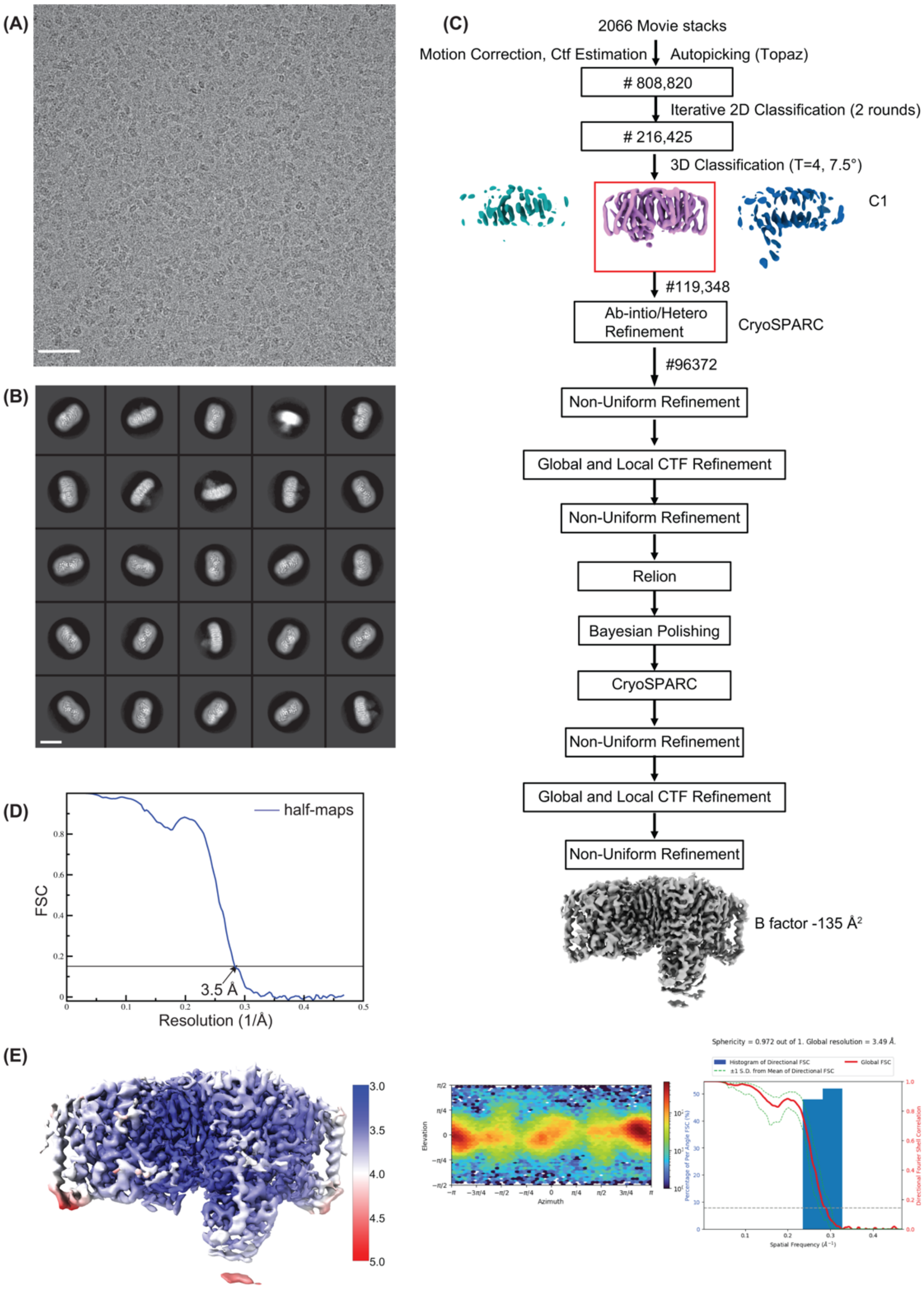

Figure S9: Cryo-EM analysis of PaMprF H566C in GDN. (A) Representative micrograph showing the enzyme in GDN micelles in ice. Scale bar is 500 Å. **(B)** Representative 2D class averages of PaMprF H566C in GDN with a box size of 320 pixels or 342.4 Å. Note that many side views reveal only one ordered soluble domain. Scale bar is 100 Å. **(C)** Image processing workflow of PaMprF H566C in GDN. As the classes and maps showed only one ordered soluble domain, the refinements and reconstructions were performed with no symmetry. Orientation distribution plot of the final PaMprF H566C map obtained from cryoSPARC represented as a heatmap. The 3D FSC plot of PaMprF H566C in GDN with C1 symmetry is shown alongside. **(D)** Fourier Shell Correlation curve of PaMprF H566C in GDN. The resolution is estimated from the FSCs by comparing the half-maps at 0.143 is ~3.5 Å. **(E)** Local Resolution of PaMprF H566C in GDN as estimated by RELION. Colour key on the right shows

the core of the enzyme is around 3.5 Å with the periphery at 4-5 Å. The figure was made with Chimera [54].

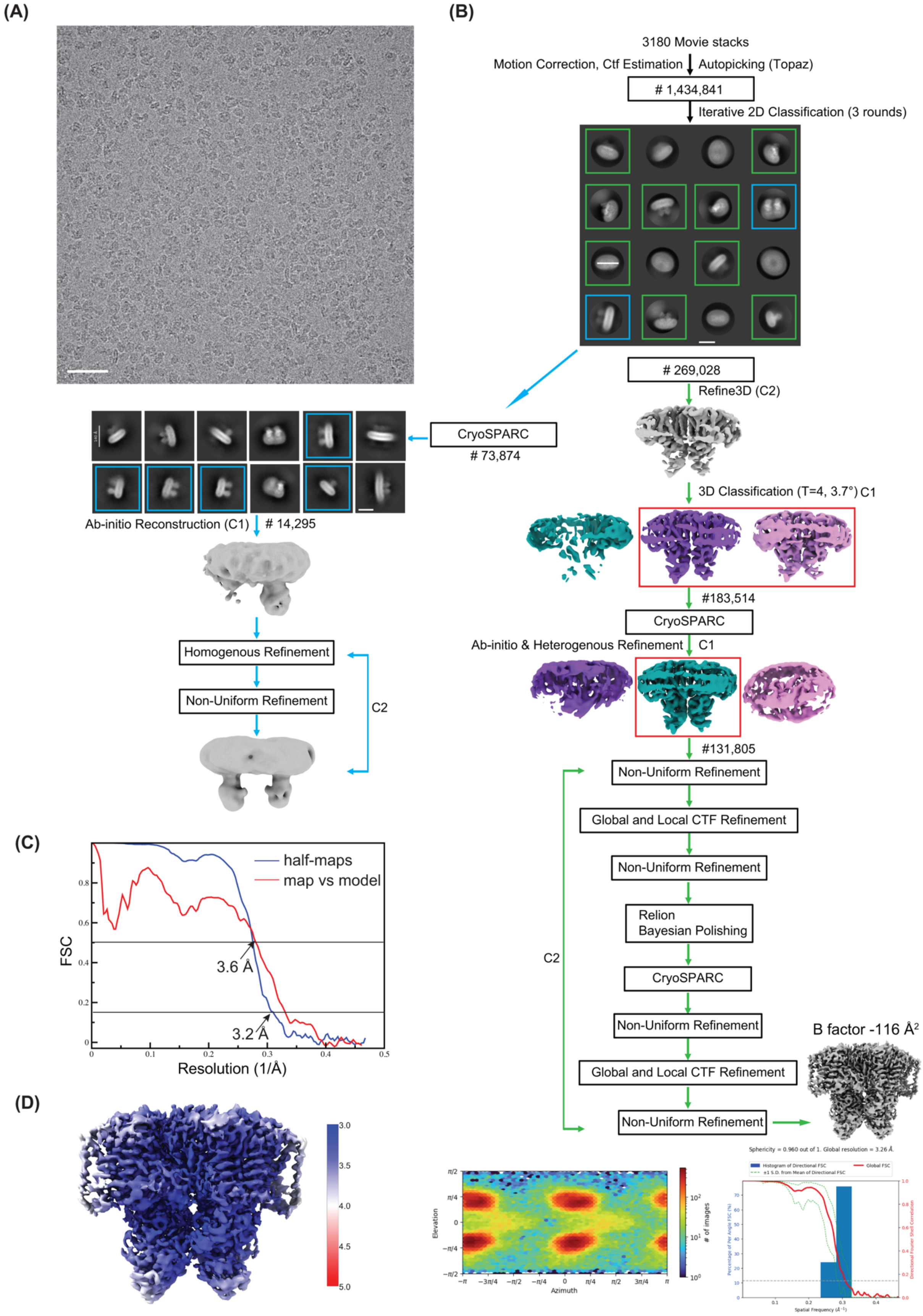
(A)
(B)
3180 Movie stacks
Motion Correction, Ctf Estimation
Autopicking (Topaz)
1,434,841
Iterative 2D Classification (3 rounds)
269,028
Refine3D (C2)
CryoSPARC
73,874
3D Classification (T=4, 3.7°) C1
Ab-initio Reconstruction (C1)
14,295
#183,514
CryoSPARC
Ab-initio & Heterogenous Refinement
C1
Homogenous Refinement
Non-Uniform Refinement
C2
#131,805
Non-Uniform Refinement
Global and Local CTF Refinement
Non-Uniform Refinement
Relion
Bayesian Polishing
CryoSPARC
Non-Uniform Refinement
Global and Local CTF Refinement
Non-Uniform Refinement
C2
B factor -116 Å²
(C)
half-maps
map vs model
FSC
3.6 Å
3.2 Å
Resolution (1/Å)
(D)
3.0
3.5
4.0
4.5
5.0
Elevation
Azimuth
# of images
Sphericity = 0.960 out of 1. Global resolution = 3.26 Å
Spatial Frequency (Å⁻¹)

Figure S10: Cryo-EM analysis of PaMprF H566C in nanodisc. (A) Representative micrograph showing the particles of PaMprF H566C in nanodisc. Scale bar is 500 Å. **(B)** Image processing workflow of PaMprF H566C in nanodisc. A sub-population of enzyme containing synthase domains farther apart (classes marked in cyan box) were processed separately (cyan arrows) to obtain a reconstruction with C2 symmetry. Due to less number of particles only a low-resolution map was obtained. The other larger population representing conventional views of PaMprF (green boxes) were processed separately with C2 symmetry to obtain high-resolution map (green arrows). Scale bar is 100 Å for the 2D classes. Orientation distribution plot of the final PaMprF H566C map in nanodisc obtained from cryoSPARC represented as a heatmap. The 3D FSC plot of PaMprF H566C in nanodisc with C2 symmetry imposed is shown alongside. **(C)** Fourier Shell Correlation curve of PaMprF H566C in nanodisc. The resolution is estimated from the FSCs by comparing the half-maps at 0.143 is around 3.2 Å (blue color) and the map vs model FSC at 0.5 is 3.6 Å (red color). **(D)** Local Resolution of PaMprF H566C in nanodisc as estimated by RELION. The core of the enzyme is around 3 Å with the periphery at 3.5 - 4 Å as shown by the colour key. The figure was made with Chimera [54].

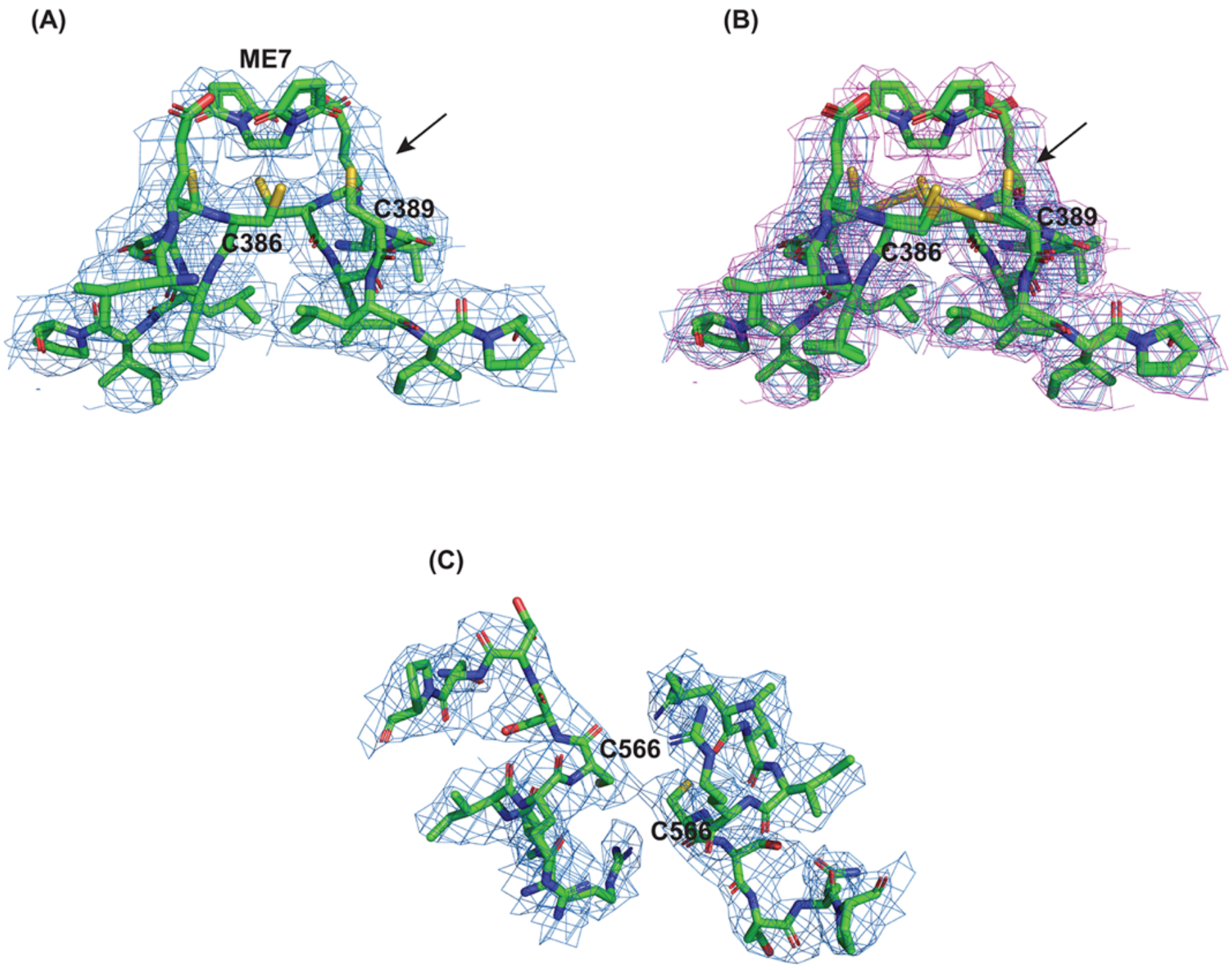

Figure S11: Cryo-EM density of select regions of PaMprF mutants. (A) An alternative model of H386C/F389C double mutant, where the density at low threshold is modelled as BMOE (ME7) and the cysteine 386 can possibly be linked with ME7. The extra density around the side chain of residue 389 (F389C) is marked with an arrow. Map was contoured at σ=4, 2 Å around the atoms with PyMOL [32]. **(B)** Overlay of the two maps of the double mutant with the two models: with disulphide between 386 and 389 and without (blue and magenta respectively, σ=6 for the blue map and σ=4 for the magenta map) showing the extra density at residue 386 indicating multiple conformers. **(C)** Density of the H566C mutant of PaMprF in nanodisc showing the cysteine residues but the disulphide bond is not modelled due to no clear connecting density (unlike the double mutant – Figure 4D). Map was contoured at σ=6, 2 Å around the atoms with PyMOL [32].

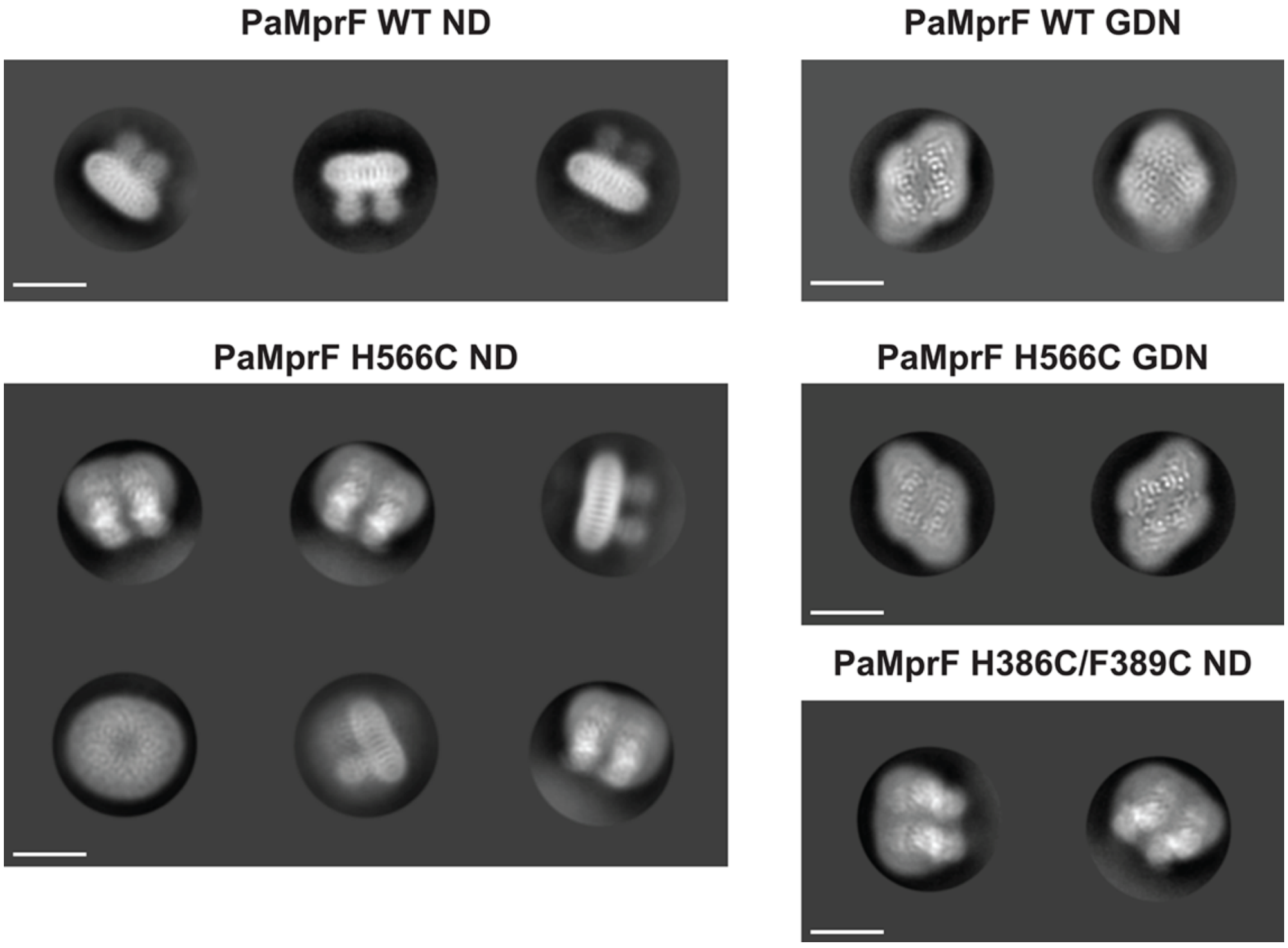

Figure S12: Unusual and less populated classes in PaMprF datasets. In a number of datasets of PaMprF that were collected, few classes observed in the 2D classification step were different from the final structure. These primarily include a class of top views corresponding to possible tetrameric assembly in the GDN datasets or side/ top tilted view of particles with the two soluble domains far apart in the nanodisc (ND) datasets. These comprise of very few particles and preferred views, typically <1-2 % of particles after 1st round of 2D classification and hence a meaningful reconstruction could not be achieved from any of these populations. Scale bar is 100 Å.

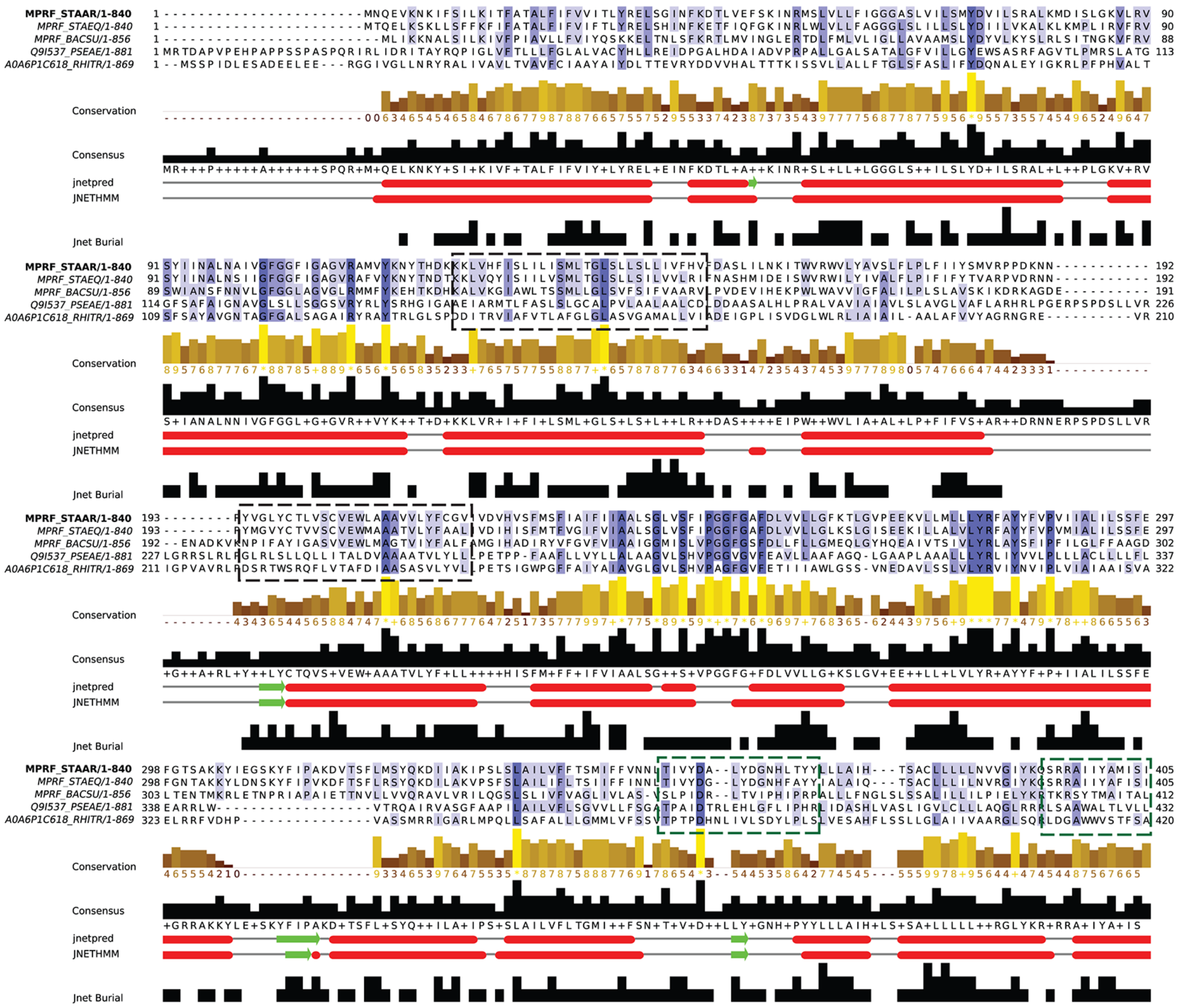
MPRF_STAAR/1-840
MPRF_STAEQ/1-840
MPRF_BACSU/1-856
Q9I537_PSEAE/1-881
A0A6P1C618_RHITR/1-869
Conservation
Consensus
jnetpred
JNETHMM
Jnet Burial

… *cont.*

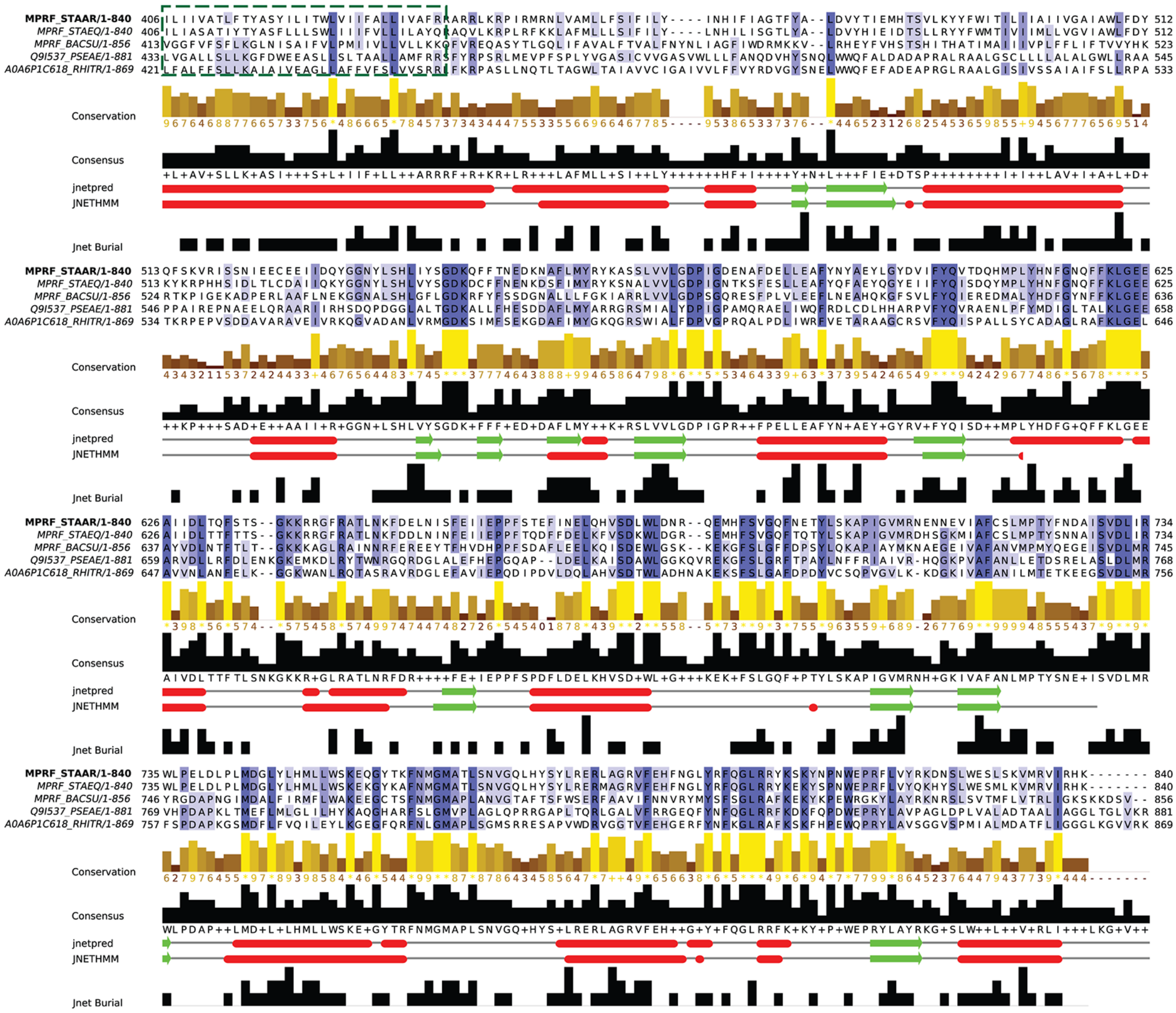

Figure S13: Sequence alignment of select MprF homologues. Select sequences of MprF homologues from uniprot were aligned with clustalW and plotted with JalView showing the conserved/consensus residues at each position, the predicted secondary structures and the propensity of residues as buried or solvent exposed. Highly conserved residues are shaded in dark blue and the regions of the enzyme that form the dimer interface in rhizobium-like (black rectangle) or Pseudomonas-like (green rectangle) are highlighted. These regions show very little conservation. The synthase domain shows higher conservation and the C-terminus of the polypeptide is predicted to be helical across the homologues. The Uniprot IDs for the sequences are: *S. aureus*, Q6GH45; *S. epidermis* Q5HPI1; *B. subtilis*, C0H3X7; *P. aeruginosa*, Q9I537; *R. tropici*, A0A6P1C618
